# Molecular Mechanisms of Chronic Pain and Therapeutic Interventions

**DOI:** 10.1002/mco2.70325

**Published:** 2025-08-07

**Authors:** Zhen Li, Xing Li, Jieqiong Liu, Rao Sun, Yingze Ye, Hongbing Xiang, Fang Luo, Shiyong Li, Ailin Luo

**Affiliations:** ^1^ Department of Anesthesiology and Pain Medicine Hubei Key Laboratory of Geriatric Anesthesia and Perioperative Brain Health and Wuhan Clinical Research Center for Geriatric Anesthesia Tongji Hospital Tongji Medical College Huazhong University of Science and Technology Wuhan China

**Keywords:** nociceptive pain, neuropathic pain, nociplastic pain, molecular mechanism, therapeutic interventions

## Abstract

Chronic pain imposes incalculable health and economic burdens, affecting more than 30% of the global population in published studies. Optimal management of chronic pain is imperative for individuals experiencing such distress. Nevertheless, the current approaches to chronic pain assessment and treatment fail to meet clinical requirements. In recent years, there has been a growing recognition of the need for precision medicine approaches to effectively manage chronic pain. Chronic pain can be classified into three categories: nociceptive (resulting from tissue injury), neuropathic (caused by nerve injury), or nociplastic (arising from a sensitized nervous system). These classifications significantly impact the evaluation and treatment decisions at all levels. Significantly, in practice, there is substantial overlap in chronic pain mechanisms among patients and within different types of chronic pain. The application of precision medicine is imperative in the management of chronic pain. This review offers a comprehensive overview of the distinctive molecular mechanisms underlying nociceptive, neuropathic, and nociplastic pain, including immune responses, ion channels, monoaminergic imbalance, and neuroinflammation. Subsequently, we summarized the status quo of nociceptive, neuropathic, and nociplastic pain manipulation. Last, we explored the advances in pain management strategies for chronic pain that are making significant progress toward their clinical implementation.

## Graphical abstract

We offer a comprehensive overview of the distinctive molecular mechanisms underlying nociceptive, neuropathic, and nociplastic pain, including the immune responses, ion channels, monoaminergic imbalance, and neuroinflammation. Subsequently, we summarized the status quo of nociceptive, neuropathic, and nociplastic pain managementmanipulation.

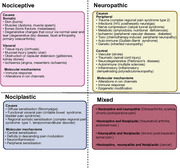

## 1 Introduction

Pain is defined by the International Association for the Study of Pain (IASP) as an unpleasant sensory and emotional experience associated with, or resembling that associated with, actual or potential tissue damage [1]. Indeed, acute pain acts as a defense mechanism against noxious stimuli and is a significant adaptive and protective mechanism under normal physiological conditions [2]. Chronic pain is defined as pain that persists or recurs for more than 3 months [3]. The global impact of this issue is substantial, making it a significant public concern due to its high morbidity rates, increased mortality rates, and significant healthcare expenses [4]. According to estimates from the recent Global Burden of Disease study, chronic low back pain (cLBP) is one of the most prevalent conditions that affects 619 million individuals worldwide in 2020; this number is expected to escalate to 843 million by 2050 [5, 6]. Among the four primary contributors to years lost to disability, three of them (namely, back pain, neck pain, and musculoskeletal disorders) are chronic pain conditions [6, 7]. The primary motivation for seeking medical care is pain, with osteoarthritis (OA), back pain, and headaches ranking among the top 10 reasons [8]. Pain relief has been a requisite and a crucial index for clinical treatment [2]. Despite advancements in therapy, the management of chronic pain remains challenging due to the progressive nature of the condition. Currently available pharmacological and nonpharmacological interventions solely provide symptomatic relief, often with limited efficacy and significant adverse effects [9]. Therefore, it is imperative to develop more effective and secure therapies that focus on addressing the fundamental causes of persistent pain.

Precision medicine pertains to the capacity of categorizing patients into distinct groups based on their varying vulnerability, biology, or prognosis associated with a specific ailment, as well as their unique reaction to a particular therapy. Consequently, treatment can be customized according to individual patient attributes [9, 10]. Applying this to chronic pain, there is a growing need to accurately classify chronic pain into its three major phenotypes (nociceptive, neuropathic, and nociplastic pain) in order to customize appropriate treatment strategies [1]. The 2021 IASP clinical criteria and grading system for nociplastic pain emphasize on the importance of early identification and accurate classification of patients based on their pain phenotype during treatment [11]. These criteria represent a crucial advancement in the pursuit of precision pain medicine, holding immense potential for the field of pain management.

Our comprehension of the phenotypic characteristics, as well as the underlying etiology and pathophysiology, of nociceptive, neuropathic, and nociplastic pain is advancing. Recent studies have further investigated the molecular mechanisms involved in chronic pain. However, in most cases, due to the absence of detailed classification of pain types and their mechanisms, the current assessment and management of chronic pain still significantly fall short of adopting a mechanism‐oriented or precision medicine strategy. This review presents an overview of the distinctive molecular mechanisms underlying nociceptive, neuropathic, and nociplastic pain, with an emphasis on the immune responses, ion channels, monoaminergic imbalance, and neuroinflammation. We delineate the status quo of nociceptive, neuropathic, and nociplastic pain management. A comprehensive overview was also given on a range of enhanced and innovative pain management strategies, including microbial intervention, nanomedicine, stem cell therapy, and gene therapy.

## 2 Overview of Subtypes of Chronic Pain

**FIGURE 1:**
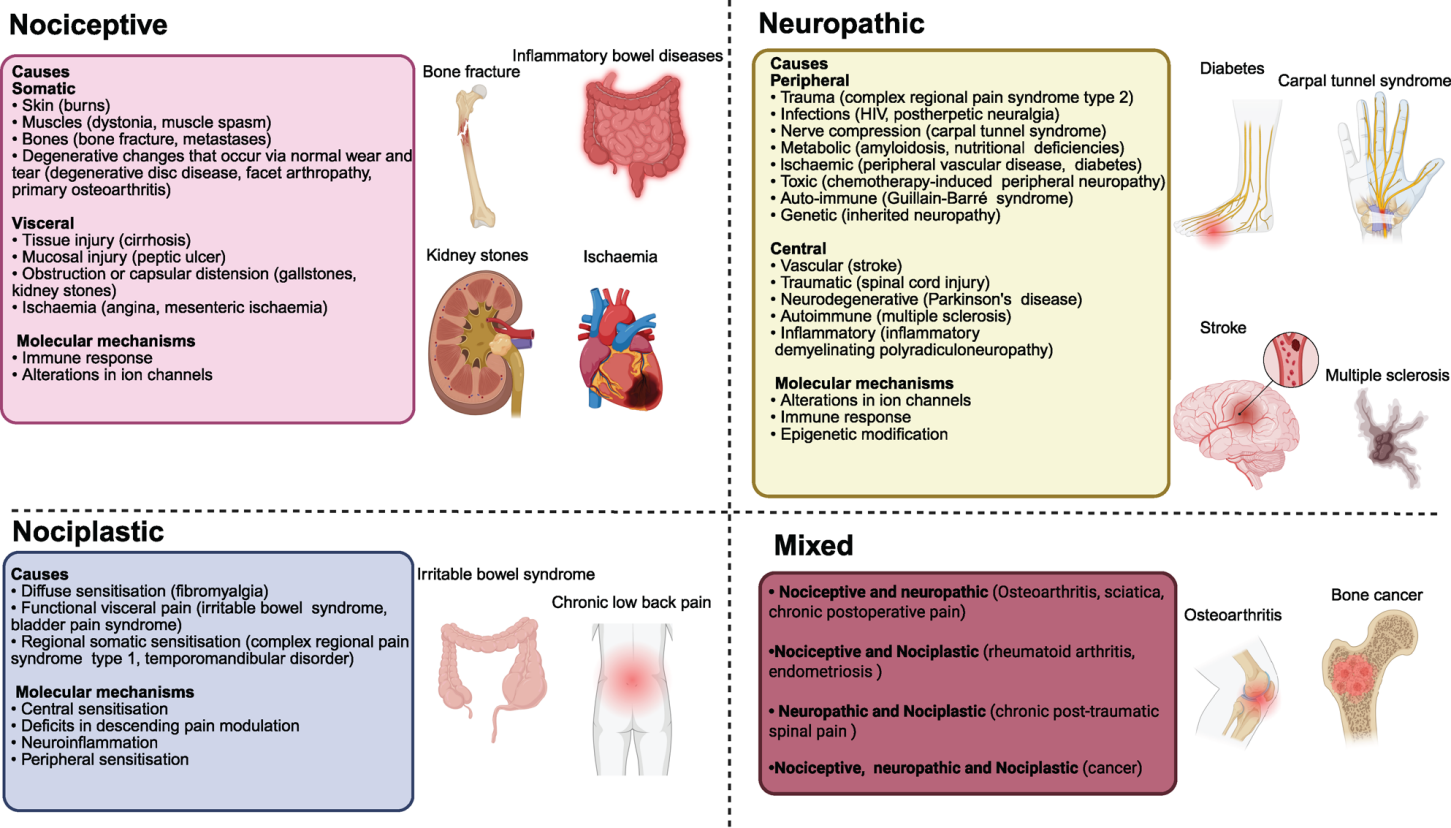
Illustrative drawing showing the various manifestations of nociceptive, neuropathic, and nociplastic pain, along with molecular mechanisms.

Chronic pain persists beyond the anticipated period of tissue healing and can be further categorized into nociceptive pain, neuropathic pain (NP), and nociplastic pain (as shown in Figure 1) [7, 12]. Nociceptive, neuropathic, and nociplastic pain result from diverse mechanisms [7]. Nociceptive pain refers to pain that results from the activation of primary afferent neurons’ peripheral terminals by noxious stimuli, or from damage or threat of damage to non‐neural tissue, and is proportional to the nociceptive input (Table 1) [2, 7, 10]. The nociceptive pain can be classified into two distinct categories: somatic nociceptive pain, which is typically localized precisely at the dermal level and characterized as pungent, lacerating, and burning; and visceral pain, which commonly manifests as a diffuse and indistinct sensation perceived in the body's mid‐line, specifically around the lower sternum or upper abdomen [13]. Chronic inflammatory pain is a form of nociceptive pain that arises from the hypersensitivity of nociceptors due to the activation of the immune system following tissue injury or infection, leading to the release of inflammatory mediators [9, 14]. Significantly, studies showed that pure nociceptive pain is rare. The longer the duration of nociceptive pain, the higher the proportion of NP [15, 16].

**TABLE 1:** Classification of chronic pain.

	Nociceptive pain	Neuropathic pain	Nociplastic pain
Cause	Tissue damage or inflammation	A lesion of disease of the somatosensory system	Maladaptive changes that impact nociceptive processing and modulation, without objective evidence of tissue or nerve damage
Examples	Trauma (e.g., burns, muscle tears, traumatic arthritis); muscle spasm; visceral pathologies (e.g., ulcers, renal stones, pancreatitis); degenerative changes that occur through normal wear and tear (e.g., degenerative disc disease, facet arthropathy, and primary osteoarthritis)	Trauma (e.g., postsurgical pain); infectious (e.g., shingles, HIV); inflammatory (e.g., acute and chronic inflammatory demyelinating polyradiculoneuropathy); nerve or nerve root compression (e.g., radiculopathy, carpal tunnel syndrome); toxins (e.g., chemotherapy); metabolic (e.g., liver disease, diabetes); ischemia (e.g., peripheral vascular disease, diabetes); hereditary (e.g., Charcot‐Marie Tooth)	Head/facial pain (e.g., migraine, chronic tension‐type headache, temporomandibular disorder); visceral pain (e.g., IBS, urological chronic pelvic pain, vulvodynia, endometriosis); musculoskeletal pain (e.g., fibromyalgia, chronic low back pain); multisensory hypersensitivity
Descriptors	Throbbing, aching, pressure‐like	Lancinating, shooting, electrical‐like, stabbing	Similar to neuropathic pain; visceral pain, might be described as diffuse, gnawing, aching, sharp
Pain pattern	Distal radiation is less common; proximal radiation is frequent around the area of the anatomical structure	Distal radiation commonly occurs in a nerve or nerve root (dermatomal) distribution	Diffuse spread is not confined to an anatomical referral pattern; patients often experience multiple nociplastic conditions.
Accompanying symptoms	Higher rates of psychopathology, including depression and anxiety, than in controls	Greater psychological distress and concomitant disability than observed in nociceptive pain	Psychological distress affects most individuals. Cognitive symptoms, insomnia, and fatigue are common. Gastrointestinal complaints and sensitivity to other sensory stimuli often occur.

Abbreviations: HIV, human immunodeficiency virus; IBS, irritable bowel syndrome.

Chronic NP refers to persistent pain resulting from a lesion or disorder affecting the somatosensory nervous system (Table 1) [17]. It can arise due to conditions like diabetes and multiple sclerosis, physical trauma caused by injury or surgery, viral infections such as Herpes and human immunodeficiency virus (HIV), as well as chemotherapy medications like paclitaxel that impact the peripheral and/or central nervous system (CNS) [18]. The pain can occur spontaneously (without any external stimulus) or be triggered by an increased response to a painful stimulus (hyperalgesia) or a painful reaction to a normally nonpainful stimulus (allodynia) [19, 20]. Common descriptors for nociceptive pain often include words like dull and pulsating, while NP is commonly characterized by adjectives such as piercing and radiating [7]. NP arises from persistent pathological changes in the functioning of the damaged nervous system. The diagnosis of NP necessitates a history of injury or disease affecting the nervous system, along with a neuroanatomically plausible distribution pattern for the experienced pain [18, 20]. In the recently published ICD11 classification, NP is categorized into either peripheral or central NP depending on whether the lesion or disease affects the somatosensory nervous system in the periphery or centrally [20, 21]. Statistically, approximately 15–25% of chronic pain is neuropathic, with the most prevalent conditions being diabetic neuropathy (DN), radiculopathy, and postherpetic neuralgia [22].

The term nociplastic pain was introduced in 2016 to delineate a distinct pain mechanism, separate from both nociceptive and NP [23]. According to the IASP, nociplastic pain is defined as “pain that arises from altered nociception despite the absence of clear evidence indicating actual or potential tissue damage activating peripheral nociceptors, or any indication of disease or lesion within the somatosensory system causing the pain” (Table 1) [23]. Nociplastic pain is characterized by diffuse body discomfort, often accompanied by fatigue and disturbances in sleep, mood, and cognition, as well as multisensory hypersensitivity. This type of pain is widely believed to be prevalent and influential in various chronic pain conditions such as fibromyalgia, low back pain, and headache. Although the precise etiology (or etiologies) of nociplastic pain remains elusive, factors such as female gender, trauma exposure, disrupted sleep patterns, early‐life stressors, heightened somatic awareness, and sedentary lifestyle contribute to an increased susceptibility to nociplastic pain [24, 25].

While the suggested definition implies that nociplastic pain is a separate category devoid of any existing or potential tissue or somatosensory harm, available evidence suggests a potential overlap of nociceptive, neuropathic, and nociplastic pain. This suggests that rather than being a distinct entity, nociplastic pain should be considered as part of a chronic pain continuum [23, 24, 25]. The term mixed pain, for instance, has been employed to characterize conditions such as cancer pain that may involve nociceptive, neuropathic, and nociplastic elements [26]. This phenomenon is observed in individuals who experience both nociceptive pain (e.g., rheumatic disorders) and NP (e.g., small fiber neuropathy), where the presence of comorbid fibromyalgia is often identified as concurrent nociplastic mechanisms [27].

## 3 Molecular Mechanisms of Nociceptive Pain Modulation

### 3.1 Inflammatory Mechanisms

Nociceptor terminals located in peripheral tissues such as the skin, joints, and gut function as detectors for essential elements of the tissue microenvironment. They are particularly adept at detecting any potentially harmful external or internal stimuli. They work synergistically with immune cells to continuously monitor tissues. However, this dual process of damage detection is characterized by the interdependent nature of immune cells and sensory neurons, which engage in intricate receptor‐ligand interactions under diverse circumstances including infections, allergies, malignancies, and tissue damage [12]. The nociceptive sensory neurons not only exhibit responsiveness to immune signals but also exert a direct regulatory influence on inflammation. For instance, nociceptors express receptors for and respond to cytokines and chemokines while also producing these inflammatory mediators [28, 29]. Consequently, it is imperative to perceive neuroimmune interactions as a synergistic and integrated catalyst and regulator of pain, thereby acknowledging nociceptive pain as a neuroimmune disorder [12].

Nociceptive pain serves as a prominent feature in various chronic inflammatory conditions, such as inflammatory bowel diseases (IBDs), implying that chronic inflammation plays an indispensable role in the pathogenesis of persistent pain [30]. In response to infection, injury, or other harmful conditions, inflammation serves as a crucial and fundamental immune reaction in organisms. This process is influenced by various factors including oxidation, carbonyl stress, C‐reactive protein (CRP), and various cytokines [31]. Inflammatory pain hypersensitivity can have an adaptive function, aiding in protecting the injured area from further damage during healing or overcoming an infection. Nevertheless, inflammatory pain has the potential to transform into maladaptive and incapacitating discomfort, such as in autoimmune disorders like OA and rheumatoid arthritis (RA), where persistent pain may occur even without ongoing clinical inflammation [12, 32]. Likewise, the presence of intestinal inflammation is widely recognized as the underlying cause of the symptoms associated with IBD [33].

#### 3.1.1 Inflammation and Pain Sensitization

The development of inflammatory pain hypersensitivity is largely influenced by the peripheral sensitization of nociceptors [12]. This occurs when transducer receptors and ion channels on the peripheral terminals of nociceptors become more easily activated in response to inflammation, leading to a decrease in their activation threshold [34]. Immune mediators produced during inflammation, including cytokines, chemokines, complement system, and antibodies, can exert their effects directly on the receptors located on the terminals of nociceptors. This leads to nociceptor sensitization through various mechanisms such as posttranslational modifications and altered membrane trafficking [12, 35].

The immune microenvironment is characterized by its diversity and dynamism, involving both tissue‐resident and infiltrating immune cells. While neutrophils are typically the first line of defense against tissue infection or injury, their absence does not appear to have an impact on acute pain hypersensitivity [36]. Mast cells, another resident myeloid population in the skin, contribute to inflammatory pain following a skin incision by synthesizing serotonin through BH4‐dependent mechanisms [37]. Recent findings have revealed that several chemokine/receptor pairs, such as CXCL1 (C‐X‐C Motif Chemokine Ligand 1)/CXCR2 (C‐X‐C Chemokine Receptor Type 2), CCL2 (C‐C Motif Chemokine Ligand 2)/CCR2 (C‐C Chemokine Receptor Type 2), CXCL13/CXCR5, and CX3CL1/CX3CR1, are secreted by dendritic cells and macrophages in the DRG and spinal cord to contribute to the development of chronic inflammatory pain [38]. Resident dendritic cells induce neuronal hyperexcitability in DRG neurons by activating the CCR4 receptor through the secretion of chemokines CCL2 and CCL17 [39]. The pronociceptive mediators IL‐1β, TNF‐α, IL‐6, prostaglandin E2 (PGE2), and reactive oxygen species (ROS) are also produced by macrophages in inflamed tissues [40]. Interestingly, macrophages can also amass in peripheral nervous tissue and transfer their mitochondria to sensory neurons, thus aiding in the alleviation of pain [41].

Suppression of complement signaling has been shown to decrease proinflammatory signaling and the release of inflammatory substances in various pain models involving rodents, such as those related to inflammation and postsurgical pain [35]. The primary soluble mediators responsible for driving inflammatory responses within the complement cascade are C3a and C5a [35]. Specific factors responsible for sensitization caused by complement activation include various local inflammatory substances, primarily released from immune cells but also from peripheral nerve endings. The mechanisms underlying the pronociceptive effects induced by complement typically involve the release of potent inflammatory molecules including nerve growth factor (NGF), calcitonin gene‐related peptide (CGRP), and IL‐1β. These molecules interact with their respective receptors on nociceptive neurons, thereby augmenting excitability and sensitizing them to both painful and nonpainful stimuli [35].

Increased NGF induced by C5a primarily promotes sensitization via the tropomyosin‐related kinase A (TrkA) receptor located on the peripheral terminals of afferent neurons [35, 42]. In situations where acute injury continues, chronic pain can be facilitated by NGF via diverse mechanisms, such as modifying the expression of ion channels involved in nociception, generating ROS, and promoting sprouting of peripheral axons [35]. NGF promotes the incorporation of Ca^2+^, K^+^, transient receptor potential vanilloid 1 (TRPV1), purinoceptor, and proton ion channels into both terminal and DRG neurons’ membranes [43, 44]. NGF also contributes to expressing acid‐sensing ion channel (ASIC) nociceptive receptors where an increase in acidity within the microenvironment caused by the accumulation of lactic acid in degenerated discs can intensify nociceptive pain [45]. All these channels are linked to ischemic and inflammatory pain, which aligns with nociceptive signaling. NGF also directly activates MAPK (mitogen‐activated protein kinase) pathway amplifying and sensitizing nerve action potentials (APs) [42]. Additionally, NGF contributes to mast cell degranulation leading to histamine release that triggers activation of nerve terminals responsible for sensing pain [46]. The pronociceptive mediator CGRP, which is produced and released by peptidergic C‐fibers during neurogenic inflammation, is also accountable for the mechanical sensitization induced by the actions of C5a in peripheral tissue. This effect could potentially involve facilitating voltage‐gated Na^+^ currents [35]. Furthermore, levels of pronociceptive cytokines and IL‐1β rise following activation of C5aR1 in a postsurgical pain model [47]. IL‐1β likely enhances Na^+^ currents through p38 protein kinase‐dependent mechanisms to heighten excitability of nociceptive neurons in different pain conditions [35].

Interferon receptor signaling in nociceptors also regulates the sensitization of nociceptors [48]. In vitro studies have shown that STING‐induced type 1 interferon reduces the excitability of human and monkey nociceptors by decreasing calcium and sodium currents [48]. However, when mice are injected with polyI:C to induce systemic interferon production, it results in heightened sensitivity to pain through MAPK‐mediated eIF4E‐dependent mechanisms. This highlights how the specific circumstances surrounding an injury determine the neuroimmune mechanisms involved in pain induction [49]. Activation of mammalian target of rapamycin by the inflammatory environment also promotes long‐term nociceptor sensitization by enhancing the terminal branching of nociceptors [50, 51].

Nociceptive sensitization is also observed in the spinal cord to facilitate the perception of pain. The activity of microglia and astrocytes is increased in patients with intervertebral disc degeneration (IDD), which contributes to a proinflammatory state by stimulating the production of cytokines and neurotrophic factors [52]. NGF also facilitates the production and backward movement of pain‐inducing neuropeptides like substance P, CGRP, and BDNF toward the dorsal horn. Moreover, prolonged inflammation leads to heightened sensitivity in afferent neurons within the dorsal horn toward peripheral nociceptor fibers [45, 53]. IL‐1β, TNF‐α, and IL‐6 also play a role in long‐term pain by influencing synaptic plasticity in dorsal horn neurons [54]. Additionally, intrathecal administration of C1q partially reverses the elevated dendritic spine density linked to peripheral inflammation while reducing inflammatory pain [55]. These findings emphasize that immune responses within tissues act as regulators for finely tuning hypersensitivity to inflammatory pain by either amplifying or inhibiting peripheral sensitization, while also prompting alterations in nociceptor structure.

#### 3.1.2 Inflammation and Nerve Growth

The innervation process is believed to be important in the development of nociceptive pain. Recent research has revealed that neurogenic inflammation through neurotrophins (NTPs) induces the ingrowth of sensory nerve fibers (hyper‐innervation) and sensitizes sensory nociceptive processing at peripheral terminals, playing an essential role in discogenic low back pain (LBP). Inflammatory cells like eosinophils, lymphocytes, macrophages, mast cells, and new microvessels serve as supplementary sources of NTPs [45, 56]. Following IDD and inflammation, there is a significant upregulation of NTPs. A direct correlation between nociceptive nerve ingrowth and NGF production by blood microvessels was observed in painful IDD [57]. Increased levels of NTPs such as NGF, BDNF, NT‐3, and NT‐4/5 are induced by the production of inflammatory cytokines IL‐1 and TNF‐α from degenerating discs. These NTPs recruit nerve fibers through activation of Trk and p75NTR receptors [45, 58]. Furthermore, pain‐related factors such as cyclooxygenase 2 (COX‐2) and nitric oxide (NO) are induced by inflammatory mediators like TNF‐α and IL‐1β, which in turn promote nerve growth [59, 60]. Tissue samples from patients with knee OA have shown the expression of NGF in subchondral mononuclear cells, chondrocytes, and osteoclasts. The expression of NGF showed a correlation with both age and synovitis scores, indicating a potential link to symptomatic OA and the experience of pain [61].

### 3.2 Alterations in Ion Channels

Receptors responsible for sensing pain possess diverse ion channels within their peripheral terminals, enabling them to detect external stimuli, encode signals, and induce membrane excitability. By regulating the current of ions across membranes, ion channels produce electrical signals. Reactive opening of voltage‐sensitive channels occurs in a sequential manner. In terms of the factors that lead to channel activation, they can be broadly classified into two distinct groups: ion channels regulated by changes in voltage and ion channels regulated by binding of specific molecules [2]. The pain sensation is transduced when harmful stimuli cause depolarization of nociceptive myelinated A‐delta (Aδ) fibers and A‐beta (Aß), as well as unmyelinated C fibers. This depolarization occurs by activation of voltage‐gated ion channels and membrane proteins located at the terminal, resulting in the conversion of these stimuli into electrical signals within neurons [29].

#### 3.2.1 Voltage‐Gated Sodium Channels

The voltage‐gated sodium channel (VGSC) family consists of nine members, namely, Nav1.1 to Nav1.9, but there still appears to be functional divergence among Nav channels. Upon depolarization of the cell membrane, VGSCs quickly transition into open conformations, enabling the influx of sodium ions (Na^+^) into cells against their concentration gradient. This mechanism triggers APs and generates nociceptive signals at nerve terminals [2]. In nociceptors’ peripheral terminals, multiple subtypes of Nav channels are engaged in transduction; specifically, somatic terminals are influenced by Nav1.7, Nav1.8, and Nav1.9, while visceral terminals are affected by the participation of Nav1.1, Nav1.6, and Nav1.9 in this role [62]. Nav1.7 and Nav1.8, which exhibit preferential expression in dorsal root ganglion (DRG) sensory neurons, play a significant role in peripheral pain signaling [63, 64].

Nav1.7 functions as a regulator of pain signaling in DRG neurons. Inflammatory agents stimulate an elevation in nociceptor activity that is dependent on Na_V_1.7, leading to an increase in the current and surface expression of Na_V_1.7 through enhanced vesicular loading, forward trafficking, and integration of Na_V_1.7 into distant axonal membranes [65]. The presence of gain‐of‐function Nav1.7 mutations results in intense pain [66], while individuals with loss‐of‐function mutations display a syndrome characterized by profound insensitivity to pain [67]. The strong association between these painful conditions and Nav1.7 mutations emphasizes the possibility of utilizing interventions aimed at modulating this channel for alleviating pain. Inhibition of Nav1.7 has also demonstrated pain attenuation in a rat model of OA, thereby providing additional evidence for the therapeutic potential of Nav1.7 blockade in alleviating pain associated with OA [66, 68]. Recent clinical trials have also shown that blocking Nav1.8 selectively can reduce chronic postoperative pain (CPPP), providing evidence that targeting peripheral Nav1.8 channels can effectively reduce pain signaling in humans [69]. Consistently, inhibiting Nav1.8 in DRG neurons could also potentially alleviate OA pain [66]. Additionally, elevated levels of the proinflammatory cytokine in colonic can activate and sensitize DRG neurons by enhancing Na_V_1.8 while suppressing voltage‐gated potassium (K^+^) currents (such as IA and IK) to enhance neuronal excitability in patients with active ulcerative colitis [33].

#### 3.2.2 Voltage‐Dependent Calcium Channels

Voltage‐gated calcium channels (VGCCs) are activated by changes in membrane potential and allow the entry of extracellular calcium ions (Ca^2+^) into cells and move down their electrochemical gradient. The presence of these channels in various types of excitable cells significantly influences cellular excitability and neurotransmission at multiple points along the somatosensory nociceptive pathways [2, 70]. They are composed of five subunits, namely, α1, α2, β, δ, and γ. VGCCs can be categorized into different types such as T‐type, N‐type, L‐type, P‐type, and Q‐type channels based on their pharmacological properties [66]. Each type has a unique combination of subunits with the α2δ subunit playing a crucial role in enhancing channel activation and inactivation rates. Increased expression of the α2δ subunit due to noxious stimuli can amplify pain signals both in the CNS and peripheral nervous system (PNS) [2].

The Cavα1 subunit and the Cavα2 subunit have been implicated with pain mechanisms specifically related to OA [71, 72]. The pain sensitivity is influenced by the Cavδ1 subunit (CACNA2D1 gene product); reducing CACNA2D1 expression in DRG neurons decreased pain sensitivity in rats suffering from OA, while increasing its expression decreased the pain threshold among healthy rats [73]. This modulation may involve alterations in CGRP secretion and modulation of the adenylyl cyclase–protein kinase A (PKA)–protein kinase C (PKC)–MAPK signaling pathway within DRG neurons [74]. Additionally, the levels of the auxiliary α2δ1 subunit were found to be elevated in male mice with arthritis, even after the subsidence of arthritis while pain persisted [75]. While gabapentin interacted with this α2δ1 subunit to block calcium flux effectively alleviates mechanical hypersensitivity during both inflammatory phases and late stages of arthritis mice models [76, 77]. These findings highlight the significance of targeting the α2δ1 subunit for managing arthritis‐induced pain behaviors [32].

Two subtypes of VGCCs have major roles to play in pain signal transmission: a T‐type Cav3.2 channel, which operates at low voltages and is involved in conducting signals along nociceptive neurons, and an N‐type Cav2.2 channel, which operates at high voltages and facilitates excitatory synaptic transmission in the dorsal horn of the spinal cord [78]. The sensory neurons of DRG exhibit a high expression level of Cav3.2 channels, which play a role in regulating the threshold for initiating APs. The involvement of Cav3.2 channels is pivotal in mediating inflammatory and NP signaling within mouse DRG neurons and dorsal horn synapses [79]. The inflammatory pain in mice is attenuated when the expression of the Cav3.2 gene is downregulated [78]. Furthermore, the pain‐relieving properties of inhibiting Cav3.2 have been confirmed in models of OA [80]. Meanwhile, Cav2.2 inhibitor also demonstrated efficacy in managing visceral inflammatory pain and osteoarthritic pain [78].

#### 3.2.3 Transient Receptor Potential Channels

Transient receptor potential (TRP) channels, extensively distributed in both the CNS and PNS, serve as highly representative ion channels activated by ligands. TRP proteins were initially recognized as a superfamily of ion channels primarily known for their permeability to Ca^2+^, but they also transport other cations such as Na^+^ and magnesium ions (Mg^2+^) [2, 66]. Mammals possess a collective count of 28 TRP channels, which can be categorized into seven subgroups according to their similarity in amino acid sequence: TRPV, TRPA, TRPC, TRPM, TRPML, TRPN, and TRPP. These channels are activated by various stimuli and play a crucial role in sensory responses to pain and stress [66]. The activation states/expression levels of TRPV, TRPM, TRPA, and TRPC show positive correlations with nociceptive pain sensation [81].

TRPV1–TRPV4 functions as nonselective cation channels that can be activated by both thermal and mechanical stimuli [82]. The TRPV1 channel, regulated by thermal stimuli and inflammatory mediators, plays a pivotal role in pain perception through its facilitation of the release of neuropeptides, such as substance P and CGRPs, from peripheral terminals [83, 84]. Decreased activity of TRPV1 has been shown to alleviate pain perception in skin conditions, OA, and internal organs [66]. The therapeutic significance of targeting TRPV1 for pain relief in OA and IBD has been underscored by clinical trials [66, 84], while the role of TRPV1 in RA‐related pain remains controversial [85]. Intriguingly, cannabinoid type 1 (CB1) paradoxically enhances the sensitivity of TRPV1 channels, leading to inhibition of AP by reducing depolarization rate in the capsaicin‐induced model [86]. TRPV4 is also implicated in the pathogenesis of chronic OA and visceral pain through its modulation of inflammatory mediators [66]. Inhibition of TRPV4 has been shown to alleviate colitis symptoms and associated pain in animal models with IBD [83]. Additionally, TRPV4 antagonist has been found to reduce knee OA‐induced mechanical hyperalgesia in rats [87, 88]. These findings indicate that targeting TRPV might provide pain relief for nociceptive pain.

The expression pattern of TRPA1 in sensory neurons and certain non‐neuronal cells such as chondrocytes suggests its potential role in the perception of pain [89]. Indeed, the involvement of TRPA1 in different types of pain, such as neuropathic and inflammatory pain, has been extensively documented [66, 83]. In particular, a recent study shows that primary sensory DRG neurons exhibit frequent spontaneous microdomain Ca^2+^ (smCa) activities, independent of APs, mediated by TRPA1 channels, and trigger continuous neurotransmission to promote chronic inflammatory pain [90]. Multiple studies have provided evidence that the pharmacological inhibition of TRPA1 using different inhibitors or gene deletion results in a decrease in both mechanical and cold hypersensitivity linked to persistent inflammation models of OA [91].

TRPM2 channels are abundantly expressed in immune cells and contribute to the regulation of inflammatory responses [66]. Exposure to H_2_O_2_ significantly enhances the expression of proinflammatory cytokines and chemokines through TRPM2 activation [92, 93]. Interestingly, inhibiting TRPM2 expression can impact the increased levels of these cytokines and chemokines [93]. Studies on TRPM2‐deficient mice have shown reduced sensitivity to thermal hyperalgesia, mechanical allodynia, and neutrophil infiltration in response to carrageenan‐induced pain [94]. Additionally, degenerated intervertebral discs display enhanced gene and protein expression of TRPC6 and TRPM2 compared with nondegenerated discs [95]. This suggests that TRPM2 may play a role in aggravating inflammatory pain. However, activation of TRPM8 selectively reduces the release of inflammatory neuropeptides while inhibiting proinflammatory cytokines’ release and leukocyte accumulation in the colon, thereby alleviating hypersensitivity [96]. Overall, these findings suggest that both TRPM2 and TRPM8 may be involved in regulating nociceptive pain mechanisms.

TRPC channels are essential for the maintenance of intracellular Ca^2+^ homeostasis by importing Na^+^ and Ca^2+^ ions while exporting K^+^ ions. These channels are responsive to various stimuli, including mechanical forces, and also exhibit voltage sensitivity. Mice lacking RPC5 or treated with the TRPC5 antagonist ML204 have shown an increase in hyperalgesia during CFA‐induced arthritis [97, 98]. Additionally, treatment with ML204 has been discovered to augment synovial inflammation and vascular swelling in CFA‐induced arthritis, indicating potential anti‐inflammatory functions of TRPC5. This highlights the possibility of targeting TRPC5 as a therapeutic approach for OA [97].

#### 3.2.4 K^+^ Channels

K^+^ channels can be classified into three main groups according to their structure and functional features: inwardly rectifying K^+^ channels (Kir), which have four subunits including the KATP channels; two‐pore domain K^+^ channels (K2P), consisting of 15 subunits; and single pore K^+^ channels K_Ca_, Kv, and Slo [66]. Activation of these ion channels results in the repolarization of the cell membrane, leading to a reduction in electrical discharge. Inflammatory agents such as cytokines, chemokines, substance P, ATP, TNF‐α, bradykinin, H^+^, and NO are recognized for their ability to promote pain by direct suppression of K^+^ conductance [99].

The Kv7 subfamily, whose members facilitate a low‐threshold noninactivating current, exerts significant influence in stabilizing the resting membrane potential, setting firing threshold, and regulating firing rate in sensory neurons. The activity of Kv7 channels is inhibited by inflammation‐induced G‐protein coupled receptors like bradykinin receptor, PGE2 receptor, protease‐activated receptor‐2, and histamine receptor [100]. This decrease in Kv7 activity mediated by inflammation is associated with an increase in nociceptor excitability and can be replicated through the utilization of targeted inhibitors like XE‐991 [101, 102]. In models with OA, a decrease in mRNA levels of Kv7 (KCNQ) channels was observed in DRG neurons, while the activation of KCNQ in this model resulted in an increase in pain threshold and a prolongation of withdrawal latency [103]. Notably, retigabine exhibits analgesic effects in models of inflammatory pain by activating peripheral Kv7 channels [104]. Flupirtine, a compound with a similar structure to retigabine, has been utilized in Europe for more than three decades to address various types of pain [105]; diclofenac is another popular anti‐inflammatory drug that engages with Kv7 channels among other targets [106].

The ability of large K_Ca_ (BK_Ca_) channel openers like NS1619 to significantly reduce mechanical hypersensitivity in CFA‐induced arthritis demonstrates that BK_Ca_ channels modulate inflammatory pain [107]. However, in this model, NS1619 does not affect thermal hypersensitivity or NP behaviors, suggesting a distinct modulatory function of BK_Ca_ channels exclusively in specific subtypes of inflammatory pain [66]. The sodium‐activated K^+^ channel Slick (K_Na_1.2, Kcnt2) in spinal cord interneurons also plays a critical role in tuning neuronal excitability in inflammatory pain [108].

#### 3.2.5 ASICs and Chloride Channels

ASICs respond to fluctuations in pH and thereby regulate synaptic plasticity and pain processing. However, tissue acidification resulting from inflammation or other neurological disorders can lead to aberrant activation of ASICs [66, 109]. Nonspecific small molecules like NS383 and amiloride have demonstrated their ability to inhibit ASIC1a and ASIC3, leading to relief in nociceptive pain caused by acid stimulation in both preclinical and clinical studies [109]. Application of the ASIC3 antagonist APETx2 has also been reported to decrease various OA pain [109, 110]. By utilizing genetically modified mice to explore the possible impact of ASIC3 on gastrointestinal function and the long‐term discomfort in the abdomen reported by individuals, scientists observed that mice lacking ASIC3 exhibited a 50% decrease in sensitivity to distention, which could be associated with reduced pain perception [111]. Furthermore, zymosan‐induced hypersensitivity in the colon was found to be attenuated in mice lacking ASIC3 [111].

A group of ASIC inhibitors that lack specificity are the commonly used nonsteroidal anti‐inflammatory drugs (NSAIDs) [112]. It is uncertain whether these drugs can directly inhibit ASICs through plasma drug levels, but they have been shown to prevent inflammation‐induced increases in ASIC expression in sensory neurons [113]. Additionally, NSAIDs like aspirin can alleviate secondary hyperalgesia by suppressing TNF‐α and/or ASIC3 in a rat model of OA [114]. Moreover, various NSAIDs have demonstrated their efficacy in reducing acid‐induced pain in human subjects [115] and inhibiting inflammation induced by acetic acid in mice [116].

#### 3.2.6 Piezo Channels

Piezo channels consist of three‐bladed propeller‐like trimers made up of 114 transmembrane helices, and they play a crucial role in the conversion of mechanical stimuli into electrical signals by cells, known as mechanotransduction [66, 117]. The permeability to cations in Piezo channels is influenced by mechanical stimuli, with Ca^2+^ having the highest permeability followed by K^+^, Na^+^, and Mg^2+^. Piezo1 is more sensitive to mechanical regulation than Piezo2 [66, 118], while Piezo2 serves as an essential channel for converting mechanical forces into electrical signals involved in touch sensation, proprioception (awareness of body position), tactile allodynia (pain caused by normally nonpainful stimuli), and mechanical pain across various types of neurons [66, 117, 119]. Under inflammatory conditions, Piezo2 mechanoreceptor plays a critical role as a mediator for touch sensitivity, suggesting that targeting this ion channel could be beneficial for treating tactile allodynia [119]. Notably, single‐cell RNA sequencing has uncovered that a particular group of nociceptors demonstrates the presence of Piezo2, suggesting its potential as a focal point for pain treatment specifically related to OA [120]. Studies involving conditional knockout have demonstrated that selectively removing or disabling the function of Piezo2 in nociceptors can provide protection against heightened sensitivity to mechanical stimulation observed in joint pain models [120].

#### 3.2.7 Purinergic Channels

Purinergic P2 receptors, which can be categorized as ionotropic (P2XRs) and metabotropic receptors (P2YRs) [121], are a type of ATP‐gated ion channels that exhibit unique characteristics and have intricate involvement in numerous physiological and pathological processes such as inflammation, perception of pain, and transmission within the neuromuscular system [66, 122]. P2XRs, such as P2X3Rs, P2X2/3Rs, and P2X7R, have been shown to have a crucial function in the maintenance of nociceptive pain. Increased concentration of extracellular ATP in inflamed tissue results in enhanced activation of P2X3 receptors in primary sensory neurons during inflammation [123]. This increased sensitivity activates the intracellular ERK signaling pathway, contributing to hypersensitivity to mechanical painful stimulation under inflammatory conditions [124]. Additionally, PGE2 amplifies P2X3R‐mediated responses through its EP3 receptor and a PKA signaling pathway [125]. Studies have shown that both antisense targeting and antagonists against P2X3R effectively alleviate hyperalgesia and mechanical allodynia in rodents with peripheral tissue inflammation [124]. Meanwhile, inhibition of P2X7R effectively suppresses the release of inflammatory cytokines, thereby exhibiting significant anti‐inflammatory properties. Research conducted on rat models with joint pain revealed a correlation between spinal ATP release and an increase in microglial P2X7R levels associated with OA‐induced pain [126]. Various animal studies demonstrated that specific inhibitors targeting P2X7R effectively alleviated both neuropathic and inflammatory types of pain [124]. These inhibitors were also explored for their potential therapeutic benefits in conditions such as RA [127, 128] and Crohn's disease [129].

Nonplatelet P2Y12 receptors located in immune cells may play a significant role in the inflammatory response [130]. In the process of neuroinflammation, activation of the P2Y12 receptor can stimulate immune cells to secrete proinflammatory cytokines, leading to increased infiltration and damage to nerve tissue and cells [131]. Moreover, the activation of P2Y12 receptors may also induce the activation of leukocytes and dendritic cells, thereby exerting a regulatory function in modulating the inflammatory response [132]. Microglia expressing the P2Y12 receptor can release various proinflammatory cytokines that contribute to pain sensation [133]. In mice with colitis, signaling through the P2Y12 receptor on spinal microglia triggers persistent visceral hypersensitivity; however, this reactivity can be prevented by inhibiting the P2Y12 receptor, which alleviates chronic pain induced by colitis as well [134]. Consequently, targeting the P2XRs and P2Y12 receptor may offer potential relief for nociceptive pain.

## 4 Molecular Mechanisms of NP Modulation

### 4.1 Alterations in Ion Channels

**FIGURE 2:**
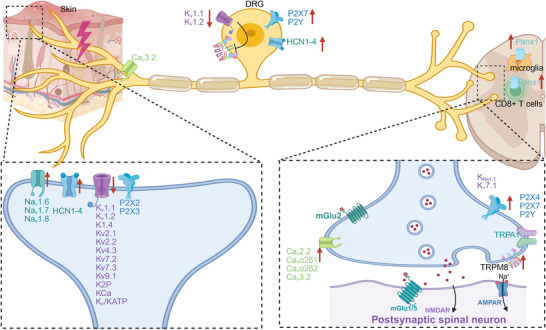
Ion‐channels involved in sensory‐spinal circuits in the development of neuropathic pain. Upregulation, increased density, and enhanced function of proexcitatory ion channels, such as Navs, TRPs, Cavs, purinergic (P2X and P2Y) channels, ASICs, and HCNs, contribute to the augmentation of neurotransmitter release, neuronal excitability, and ectopic firing in peripheral sensory neurons, DRG, and spinal dorsal cord. This is further enhanced by the downregulation of potassium channels, such as the diminished inhibition of the TRPM8 due to decreased potassium channels in DRG. Cavs indicates voltage‐gated calcium channels; DRG, dorsal root ganglion; HCNs, hyperpolarization‐activated cyclic nucleotide‐gated channels; Navs, voltage‐gated sodium channels; TRPs, transient receptor potential channels; TRPM8, transient receptor potential (TRP) transient receptor potential melastatin 8. The red arrow signifies an increase or decrease, while other‐colored arrows indicate secretion.

Different types of noxious stimuli to the nerve are transduced with remarkable specificity by a range of ion channels from the TRP family, alongside ASIC channels or purinergic channels. Subsequently, the activation of VGSCs serves to enhance the amplitude of TRPs and induce depolarization levels sufficient enough to initiate APs. On the other hand, hyperpolarization caused by diverse types of Kv channels can block these transient potentials effectively. Finally, VGCCs located at nerve endings play a crucial role in the release of neurotransmitters by facilitating the fusion of vesicles. Recent literature has also found that pannexin‐1 channels on microglia and CD8^+^ T cells differentially contribute to NP through vascular endothelial growth factor (VEGF) and leptin [135]. It is not surprising that all these ion channel types undergo strict regulatory control through posttranslational modifications and transcriptional regulation processes, which may become deregulated following nerve injury (as shown in Figure 2) [21].

#### 4.1.1 Voltage‐Gated Calcium Channels

VGCCs are strongly linked to the development of NP, particularly through the α2δ subunit. Their biophysical properties allow for the determination of neuronal activation and rhythmicity, making them highly “druggable” targets in various neural disorders, including chronic pain [70]. In addition to modulation of Ca^2+^ influx, α2δ subunit modulates neuronal excitability by enhancing its expression and localization via interaction with other VGCC‐associated subunits. The α2δ subunit is extensively targeted for managing NP using antiepileptic drugs such as gabapentin and pregabalin [21, 70]. Each individual α2δ subtype—α2δ1, α2δ2, and α2δ3/4—has its distinct role in NP [70].

The expression of Cavα2δ1 in sensory neurons is increased following injury to peripheral nerves, leading to enhanced transportation of Cavα2δ1 to the axons of central presynaptic terminals in the dorsal sensory neurons of the spinal cord [136, 137]. Activation of the thrombospondin‐4 (TSP4)/CaVα2δ1‐dependent pathway following peripheral nerve injury can excessively enhance excitatory synaptic input and cause hyperexcitability and excitatory synaptogenesis in sensory neurons via the PKC signaling pathway, thereby contributing to central sensitization and pain states [138]. α2δ1 can also promote the delivery of N‐methyl‐D‐aspartic acid receptors (NMDARs) and modulates the intracellular assembly of postsynaptic Ca^2+^‐permeable a‐amino‐3‐hydroxy‐5‐methyl‐4‐isoxazolepropionic acid receptor [139, 140]. Meanwhile, α2δ1 is responsible for guiding Cav2.2 to the cell surface of DRG neurons and directing it toward the presynaptic terminal in the dorsal horn [141]. Reducing the expression of α2δ1 leads to decreased activity of presynaptic terminal Cav2.2, resulting in reduced glutamate release that impacts nerve signaling. BK_Ca_ channels possess a distinct extracellular N‐terminal region that interacts with α2δ1, thereby inhibiting excessive neuronal excitation [2]. Following peripheral nerve injury, there is a notable decrease in the expression of large conductance BK_Ca_ channels. This reduction alleviates the inhibition on the α2δ1 subunit, facilitating the transportation of Cav2.2 channel to the presynaptic site and exacerbating NP [70, 142]. The interplay between these subunits plays a crucial role in maintaining normal nervous system function and pain transmission mechanisms.

α2δ2 subunit modulates the activity of calcium channels in both the PNS and CNS through its interaction with calcium channels, particularly N‐type and P/Q‐type calcium channels [70]. α2δ2 subunit enhances calcium channel activity, leading to increased glutamate release [143]. In addition, the presynaptic calcium channel α2δ2 not only modulates the levels and functionality of GABA_A_ receptors in the postsynaptic region, but also exerts an influence on the internal dynamics, effectiveness, and durability of these receptors. As a result, it significantly affects the process of NP [144]. The role of α2δ3 may be associated with the regulation of neuronal excitability related to pain and involved in the transmission and processing of central pain signals. Conversely, despite being a subunit of calcium channels, α2δ4 appears to have minimal contribution to the onset and advancement of NP [70].

The levels of Cav3.2 expression are elevated in various experimental models of traumatic nerve injury, along with an increase in the amplitude and density of T‐type calcium channel currents [21]. A Cav3.2 inhibitor has been found to prevent paclitaxel‐induced neuropathic allodynia and spontaneous activity in sensory neurons [145]. The study also proposed that there may be interactions between Cav3.2 and Toll‐like receptor 4 (TLR4) signaling pathways, both of which have been associated with nociceptive sensitization.

#### 4.1.2 TRP Channels

The association between NP and TRPA, TRPM, and TRPV channels has been demonstrated in previous studies [146]. In patho‐/physiological conditions, mechanical stimuli can partially activate TRPA1 and TRPV4; cold temperatures activate TRPA1 and TRPM8; while hot temperatures and acidic pH activate TRPV1 [147, 148]. Following nerve injury or neuropathic conditions, ROS/reactive nitrogen species released during cell damage directly stimulate the activation of TRPA1 [147, 148]. TRPM2 can be triggered by different stimuli, including adenine dinucleotide, ROS, and intracellular Ca^2+^ [149]. Both nerve damage/injury and an enhanced inflammatory microenvironment upregulate the expression of these sensory dominant TRP channels, leading to heightened excitability of nerve fibers in terms of magnitude and duration [147]. Numerous studies utilizing genetically modified mice lacking specific functional TRP channels or employing pharmacological blockers for individual ones have consistently demonstrated their significant contribution to the stimulation of peripheral nerve fibers as well as NP‐related behaviors observed in rodent models [147, 148].

TRPA1 expressed in Schwann cells facilitates pain sensation induced by CGRP and capsaicin in rodents [150]. TRPA1 antagonists showed some effectiveness in rats with milder neuropathy, indicating its role in moderating milder neuropathy [151]. Additionally, studies have shown an enrichment of rare variants of the TRPA1 gene among individuals suffering from chronic neuropathic and nociplastic pain conditions [152]. Research conducted on mice has suggested that the presence of TRPM2 in macrophages and spinal glial cells exacerbates inflammatory signals linked to pain, thereby impacting the pathophysiology of inflammation and NP [94, 146, 153]. Additionally, inhibition of TRPM3 has been shown to alleviate oxaliplatin‐induced peripheral NP in mice [154]. On the other hand, inhibition of TRPM8's effects appears to alleviate pain in chemotherapy‐induced NP (CINP), migraine, and trigeminal neuralgia [155, 156, 157]. Therefore, these results indicate that focusing on targeting TRPA1 and TRPM could offer a novel approach to managing NP.

The TRPV1 channel serves a dual purpose in NP by functioning as a “pain regulator” through its sensitization and desensitization processes. The contribution of TRPV1 to mechanisms linked to NP arises from its interaction with cytokines, ion channels, neurotransmitter signaling, and oxidative stress. The formation of a sensitive signaling complex between TRPV4 and the small GTPase RhoA further contributes to adverse alterations in cellular morphology in the context of neurological injuries and diseases [158]. Moreover, activation of TRPV4 initiates TRPV4‐dependent microgliosis, which leads to enhanced neuronal excitability and dendritic spines remodeling through lipocalin‐2 derived from microglia in response to nerve injury [159]. Pathological variations in the TRPV4 gene can result in ion channel dysfunction leading to axonal neuropathies such as spinal muscular atrophy [160]. From a therapeutic standpoint, capsaicin, which acts as a TRPV1 agonist, can induce analgesia by causing receptor desensitization [161]. Therefore, targeting TRPV with medications could potentially mitigate these detrimental effects and offer novel therapeutic options for neurological conditions that currently lack efficacious therapies.

#### 4.1.3 Acid‐Sensing Ion Channel

It has been shown that ASIC3−/− mice exhibited similar development of mechanical and heat hyperalgesia to WT mice following spinal nerve ligation, suggesting a limited involvement of ASIC3 in NP [162]. However, the involvement of ASIC3 in NP has been further confirmed by the observed upregulation of ASIC3 expression in DRG neurons, concomitant with mechanical hyperalgesia, which was ameliorated by administration of the ASIC inhibitor amiloride in mice with NP [163]. Similarly, the expression of ASIC3 gene is upregulated following nerve injury in DRG neurons, and deletion of ASIC3 results in shortened duration and reduced intensity of mechanical and thermal hyperalgesia in chronic NP [164]. Moreover, by modulating macrophage polarization and transcription factor expression in NP, deletion of ASIC3 gene alleviates mechanical allodynia and attenuates thermal hyperalgesia [165]. Collectively, these studies provide compelling evidence for the role of ASIC3 in peripheral nociceptive perception, thus identifying this channel as a promising target for pain management [109].

#### 4.1.4 Purinergic Channels

The involvement of P2 purine receptors, including P2X3, P2X2/3, P2X4, P2X7, and P2Y in NP has been demonstrated [166, 167]. The P2X3 and P2X2/3 receptors exhibit high expression levels in neurons of the DRG and nodose ganglion [168]. Following tissue injury, ATP release activates the P2X3 and P2X2/3 receptors present on peripheral primary sensory nerve fibers [169], potentially serving as the initiating factor for NP. Furthermore, studies have demonstrated that knockdown of P2X3 using antisense techniques can effectively reverse mechanical allodynia in NP models. On the other hand, P2X4 and P2X7 receptors are predominantly expressed in microglia [170]. Upon nerve injury, ATP activation of P2X4 and P2X7 receptors induces the opening of ion channels in microglial cell membranes, leading to an influx of Na^+^ and Ca^2+^, efflux of K^+^, and subsequent release of inflammatory factors. Consequently, this cascade leads to an increase in aberrant neuronal activity and heightened synaptic transmission [166]. Studies have indicated that P2X4 or P2X7R antagonists can inhibit the release of inflammatory factors and alleviate NP further supporting its role in this condition [171, 172].

P2Y receptors distributed across neurons and glial cells play a crucial role in regulating neurotransmission. In the context of nerve injury, P2Y receptors are activated and upregulated in satellite glial cells (SGCs) and neurons through diffusible nucleosides/nucleotides. The activation of SGCs and neurons triggers the secretion of cytokines and chemokines that mutually stimulate each other and modulate NP, thereby causing peripheral amplification [148, 167, 173]. Within the CNS, diffused cytokines, chemokines, and BDNF cause glutamate release from the presynaptic domains while also enhancing the binding affinity between glutamate and its receptors on postsynaptic domains. Numerous studies have consistently confirmed a robust positive correlation between the elevated expression level of P2Y receptors and the generation of NP [167, 173]. Additionally, several reports have shown that P2Y short hairpin RNA or P2Y antagonists can serve as analgesics by reducing P2Y receptor expression levels and neural cell activation to alleviate NP [174, 175]. Therefore, future studies should focus on investigating the viability of gene therapy and P2Y receptor antagonists for treating NP.

#### 4.1.5 Voltage‐Gated Sodium Channels

Due to the excitability of nerves caused by nerve damage, Nav channels are considered the primary molecular entities involved in NP conditions. Generally, increased expression of Nav channels has been linked to a decrease in the activation thresholds of nociceptors and the occurrence of aberrant activity. In various rodent neuropathic models, there has been evidence of increased expression, trafficking, and localization to the peripheral regions of several Nav channel isoforms. These include Nav1.3 and Nav1.6 on myelinated axons, as well as Nav 1.7 and Nav 1.8 on unmyelinated axons [148, 176, 177]. Furthermore, numerous investigations employing mouse genetics and pharmacological treatments aimed at Nav channels have substantiated their role in stimulating peripheral nerve fibers and behaviors associated with NP [148, 176, 177]. Recent studies have also implicated sodium channels in human NP disorders. The contribution of Nav1.7 channels in sensory neurons to the development of NP has been established. Enhanced pain in painful diabetic neuropathies has been associated with genetic variations in Nav1.7 [178], and the anomalous enhancement of resurgent Nav1.7 currents at high temperatures, caused by SCN9A mutations, underlies the episodic heat‐enhanced pain in inherited erythromelalgia [179]. Moreover, the intranasal administration of a CRMP2–Ubc9 inhibitor has been shown to regulate Nav1.7 activity and effectively alleviate trigeminal NP [180]. Similarly, mutations in Nav1.8 channel predominantly expressed in peripheral nociceptive neurons, have been associated with painful small‐fiber neuropathy [181].

#### 4.1.6 K^+^ Channels

In various rodent models of NP, there has been a decrease observed in the expression levels of several K^+^ channel proteins such as Kv1.1, Kv1.2, K1.4, Kv2.1, Kv2.2, Kv4.3, Kv7.2, Kv7.3, and Kv9.1, along with some K2P, K_Ca,_ K_Na_1.1, and Kir/KATP channels which ultimately leads to decreased K^+^ currents and increased excitability of sensory nerves causing hyperexcitation phenomenon to occur within them [182, 183]. The expression of KCNA2 (encoding 1.2) and KCNS1 (encoding 9.1) was found to be downregulated in the DRG of rats with spinal nerve ligation, whereas their overexpression has been demonstrated to alleviate pain hypersensitivity [184, 185]. In line with this, inhibition of KCNA2 or KCNS1 contributes to NP in rats by reducing Kv current and increasing excitability in the DRG [185, 186]. Furthermore, activating KCNQ (encoding Kv7) channel using retigabine reduced mechanical allodynia and thermal hyperalgesia in diabetic rats, while inhibition of KCNQ with XE991 intensified pain‐related behaviors [187]. SUMOylation of neurons’ Kir7.1 also contributes to NP by regulation of its membrane expression in spinal cord [188]. Interestingly, inhibitory current IKD generated by Kv1.1–1.2 channels serves to counterbalance the activity of TRPM8 in determining cold sensitivity [21, 189]. Of course, further investigation is required to thoroughly examine the impact of modified expression and/or functionality of various K^+^ channels in neuropathic conditions using both pharmacological interventions and mouse genetic studies [148].

#### 4.1.7 Hyperpolarization‐Activated and Cyclic Nucleotide‐Gated Channels

Hyperpolarization‐activated and cyclic nucleotide‐gated (HCN) channels represent another group of excitatory channels that have been strongly associated with NP. All peripheral sensory neurons express the four known HCN channels, with HCN1 and HCN2 playing a particularly crucial role in generating an inwardly‐rectifying current (Ih) through hyperpolarization activation in sensory neurons [21, 190]. The levels of HCN1 and HCN2, along with the current Ih, show a substantial increase in CNS sensory neurons in rodent models of NP [21, 191]. Conversely, the use of drugs like ivabradine and ZD7288 to block these channels has been shown to reduce neuropathic hypersensitivity in rodents with neuropathy [192]. Additionally, elimination of HCN2 specifically in Nav1.8‐expressing nociceptors resulted in a complete absence of neuropathic mechanical and thermal allodynia in mice [191]. Activation of HCN channel in NP is modulated by cyclo‐oxygenase‐2 through spinal NMDAR [190, 193]. Certain drugs with additional HCN‐blocking activities, such as eugenol, dexmedetomidine (α2 agonist), propofol, minocycline, and methylcobalamin, have also demonstrated pain‐alleviating effects in various pain models [190].

### 4.2 Immune Mechanisms

**FIGURE 3:**
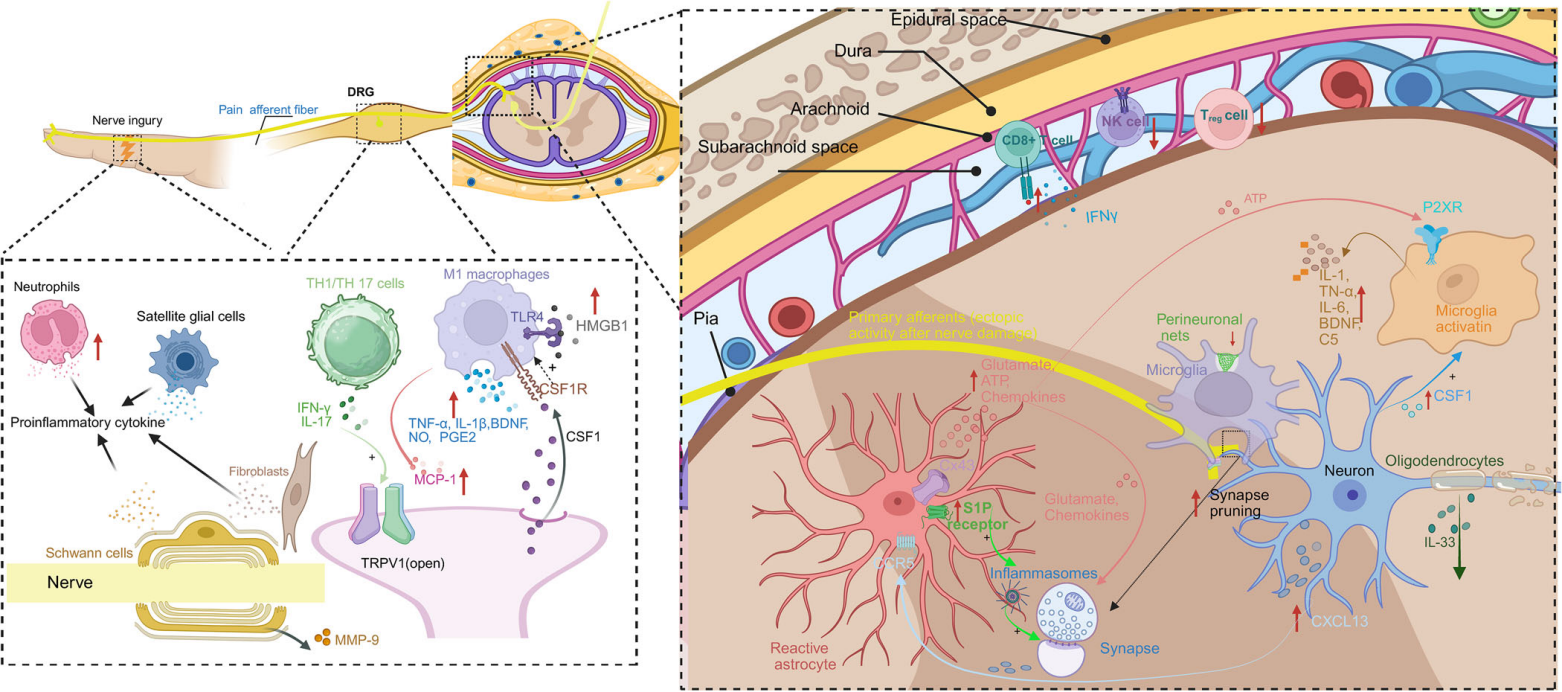
Interactions between nociceptive neurons and immune cells implicated in the development of neuropathic pain in peripheral nerves, DRG, and CNS. Neutrophils and proinflammatory macrophages are rapidly recruited to sites of nerve injury or damage, as well as DRG, and play a role in the development of NP by proinflammatory mediators, including TNF‐α, IL‐1β, BDNF, NO, and PGE2. Macrophages in the DRG are essential for initiating and maintaining mechanical allodynia induced by nerve injury through interaction between axotomized CSF1^+^ sensory neurons and CSF1 receptor‐positive macrophages. Additionally, there is an increase in macrophage numbers dependent on TLR4‐ and MCP‐1‐mediated mechanisms Similarly, fibroblasts produce proalgesic mediators such as NGF and IL‐6 that drive sensitization of peripheral neurons to promote neuropathic pain. The pain‐inducing effects of effector T cells are ascribed to the proinflammatory T_H_1 and T_H_17 subtypes, which produce their respective effector cytokines IFNγ and IL‐17. T cell‐derived cytokine IL‐17 directly activates TRPV1 nociceptors, thereby contributing to the induction of mechanical allodynia. The Schwann cells and SGCs play a significant role in pain sensation after nerve injury through the release of various inflammatory mediators. Furthermore, the blood–nerve barrier is disrupted by Schwann cells through the release of MMP‐9. Peripheral nerve injury induces an upregulation of TNF‐α, and BDNF, components signaling in microglia within the CNS. Microglia also degrade the perineuronal nets and prune inhibitory interneuron synapses in the dorsal horn, thereby contributing to NP. Following nerve injury, astrocytes experience a decline in their capacity to regulate the balanced levels of extracellular K+ and glutamate due to increased Cx43, thereby causing an increase in neuronal excitability. Furthermore, nerve injury induces the upregulation of CXCL13 in spinal cord neurons, thereby activating astrocytes through CCR5 signaling pathway to sustain neuropathic pain. The injury to the nerves also triggers an upregulation of SP4 in spinal cord and cortical astrocytes, thereby facilitating neuropathic pain through the generation of new synapses and reorganization of somatosensory cortical circuits. In addition, the activation of S1P receptor found on spinal astrocytes triggers the activation of inflammasomes. Finally, IL‐33 derived from oligodendrocytes may act on spinal microglia, astrocytes or endothelial cells to facilitate synaptic transmission. BDNF indicates brain‐derived neurotrophic factor; CCR5, chemokine (C‐C motif) receptor 5; CNS, central nervous system; CSF1, colony stimulating factor 1; Cx43, connexin‐43; CXCL13, chemokine (C‐X‐C motif) ligand 13; IFNγ, interferon gamma; IL‐6, interleukin 6; IL‐17, interleukin 17; IL‐1β, interleukin‐1 beta; IL‐33, interleukin 33; NGF, nerve growth factor; TNF‐α, tumor necrosis factor alpha; TLR4, toll‐like receptor 4; MCP‐1, monocyte chemoattractant protein 1; MMP‐9, matrix metalloproteinase‐9; NO, nitric oxide; NP, neuropathic pain; PGE2, prostaglandin E2; S1P, sphingosine‐1‐phosphate; SGCs, satellite glial cells; TRPV1, transient receptor potential vanilloid 1; TSP4, thrombospondin‐4. The red arrow signifies an increase or decrease, while other‐colored arrows indicate secretion. The plus sign (+) signifies promotion.

Neuroinflammation linked to NP arises from reciprocal communication between immune cells and nociceptive neurons following injury in the somatosensory nervous system [194]. Similar to immune cells, the nociceptive neurons possess cytokines, chemokines, and TLR that play a crucial role in immune regulation. Injury to the nervous system triggers the release of cytokines, chemokines, and proinflammatory agents. These substances activate resident glial and immune cells while attracting circulating leukocytes toward the injury site as well as various regions along the neural pain axis (including DRG, spinal cord, and supraspinal brain areas). Multiple types of immune cells have been involved in neuroinflammation such as neutrophils, macrophages, dendritic cells, fibroblasts, T‐cells, B‐cells, and glial cells [12, 28, 195]. The release of inflammatory agents by these cells includes TNF, alpha diverse IL, ROS/NOS, bradykinin, NGF, and PGE2 which heighten neuronal excitability while suppressing inhibitory pathways [12, 195, 196]. These alterations result in sensitization of the somatosensory signaling pathway leading to the development of symptoms associated with NP [12, 195]. Therefore, disturbances between immunity and neuronal activity throughout all stages of pain processing from peripheral regions to cortical areas largely contribute to pain perception (as shown in Figure 3) [12, 195].

#### 4.2.1 Peripheral Neuroinflammation

Numerous immune cells of both innate and adaptive nature can be found within damaged peripheral nerves and DRG, contributing to NP. Neutrophils are swiftly attracted to locations where nerve harm or injury has occurred, as well as DRG, and play a role in the development of NP [28, 195]. Inflammatory macrophages are attracted to areas of nerve damage and DRG, where they secrete significant quantities of proinflammatory mediators, including TNF‐α, IL‐1β, BDNF, NO, and PGE2. These mediators enhance nociceptive signaling and contribute to the development of NP [195]. Macrophages in the DRG are essential for initiating and maintaining mechanical allodynia induced by nerve injury by means of communication between axotomized sensory neurons expressing colony stimulating factor 1 (CSF1) and macrophages that possess the CSF1 receptor [195, 197]. The CX3CR1^+^ macrophages undergo proliferation and expansion in response to peripheral nerve injury in mice within the DRG [198]. Additionally, there is an increase in macrophage numbers within the DRG in rats with CINP; this causes mechanical allodynia dependent on TLR4‐ and monocyte chemoattractant protein 1 (MCP‐1)‐mediated mechanisms [199]. Similarly, fibroblasts produce pronociceptive mediators like NGF and IL‐6 that drive sensitization of peripheral neurons to promote NP [200, 201]. Fibroblasts located within the meningeal sheath surrounding the DRG as well as epineurium/perineurium surrounding an injured nerve also expand their population size while secreting protease inhibitor PI16; this enhances the sensitivity to pain by inducing an elevation in vascular permeability along with leukocyte infiltration into affected areas [12, 202]

The infiltration of T cells into damaged peripheral nerves, DRG, and spinal cord following nerve injury contributes to the maintenance of pain hypersensitivity. Additionally, reducing CD4^+^ cell levels alleviates mechanical hypersensitivity. The pain‐inducing effects of effector T‐cells are ascribed to the proinflammatory T_H_1 and T_H_17 subtypes, which produce their respective effector cytokines IFNγ (interferon gamma) and IL‐17. In a similar manner, the pain hypersensitivity that arises from paclitaxel treatment is intensified by the transfer of CD8^+^ T‐cells. Conversely, when the function of CD8^+^ T cells is hindered using neutralizing antibodies, mechanical allodynia is alleviated [12, 195, 196]. The activation of TRPV1 nociceptors, which are responsible for the development of mechanical allodynia in a CINP model, is directly facilitated by IL‐17, a cytokine derived from T‐cells [203]. The Schwann cells and SGCs, which are the major glial cells in the PNS, play a significant role in pain sensation after nerve injury. The activation of these peripheral glial cells precedes that of central glia in response to painful stimuli, leading to the release of various inflammatory mediators. This process sensitizes nociceptors on Schwann cell axons and SGC cell bodies [204]. Furthermore, the blood‐nerve barrier is disrupted by Schwann cells that have been activated. This disruption occurs through the release of matrix metalloproteinase‐9 (MMP‐9), which facilitates the attraction of immune cells from blood vessels. Subsequently, these immune cells release additional pronociceptive mediators following nerve injury. [204]

#### 4.2.2 Meningeal Immune System

The meninges enveloping the brain and spinal cord comprise the dura mater, pia mater, and arachnoid mater, which house immune cells and contribute to both immune surveillance and neural function regulation [205, 206]. The recent research findings have revealed the strong correlation between the peripheral immune system and the CNS, along with the possible involvement of meningeal immunity in pain conditions [195, 207]. Intrathecal administration of CSF1, even without injury, can cause pain behavior in male mice through activation of microglia within the spinal cord meninges [208]. Meanwhile meningeal Treg cells in female mice potentially enhance their suppressive effect on CSF1‐induced immune activation by releasing cytokines and chemokines that modulate microglial function [208]. An increased occurrence of NK cells in the cerebrospinal fluid (CSF) was also found to be linked with reduced susceptibility to mechanical pain among individuals diagnosed with herpes zoster neuralgia or polyneuropathy. This negative association implies that NK cells may have a safeguarding impact on the persistence of pain [209]. Increased frequency of CD8^+^ cells in the CSF was found to be associated with heat hyperalgesia and heightened sensitivity to mechanical pain in patients suffering from herpes zoster neuralgia [209]. However, it was observed that the frequency of CD8^+^ cells showed an inverse correlation with mechanical pain sensitivity in patients with polyneuropathy [209], suggesting a potential protective effect of CD8^+^ cells in certain chronic NP syndromes [195]. These findings collectively suggest that modulating immune function in the meninges shows potential as a viable therapeutic strategy for the treatment of NP.

#### 4.2.3 Central Neuroinflammation

The resident myeloid cells in the CNS, known as microglia, play a crucial role in NP. While playing a crucial role in the process of repairing and remyelinating after an injury [210, 211], microglia also undergo proliferation and activation following damage to peripheral nerves. This occurs due to signaling triggered by injured neurons releasing CSF1 that interacts with microglial receptors known as CSF1R. Consequently, this interaction contributes to the development of pain [197]. Peripheral nerve injury induces an upregulation of TNF‐α and BDNF signaling in the microglia within spinal cord and hippocampus, leading to synaptic modifications and NP [212]. The mechanical hypersensitivity induced by nerve injury can be alleviated through the deletion of BDNF in CX3CR1^+^ microglia or selective ablation of microglia [213]. Additionally, following peripheral nerve injury, spinal cord microglia exhibit significant upregulation of the components involved in the terminal complement cascade, namely, C5 and C5aR1. Administration of a potent and selective antagonist targeting C5aR1, PMX53, via intrathecal route effectively mitigated NP [35]. Spinal microglia are also capable of pruning inhibitory interneuron synapses, thereby contributing to NP through an excitatory‐inhibitory imbalance [214]. In addition, microglia degrades the perineuronal nets that surround spino‐parabrachial neurons within the spinal dorsal horn, thereby contributing to the development of NP following nerve injury [215].

Following nerve injury, astrocytes experience a decline in their capacity to regulate the balanced levels of extracellular K^+^ and glutamate, thereby causing an increase in neuronal excitability [216]. Additionally, there is a sustained increase in connexin‐43 (Cx43) expression in astrocytes after nerve injury, leading to a shift in its role from facilitating gap junction communication to modulating cell‐to‐cell signaling through paracrine mechanisms [217]. The paracrine regulation results in an augmented release of glutamate, ATP, and chemokines, which function as neuromodulators and have the ability to enhance the transmission of excitatory synapses in the spinal cord's pain circuitry [217, 218, 219]. Furthermore, nerve injury induces the upregulation of CXCL13 in spinal cord neurons, thereby activating astrocytes through CCR5 signaling pathway to sustain NP [218, 219]. Thus, the facilitation of NP by chemokines is mediated through bidirectional interactions between neurons and astrocytes. The injury to the nerves also triggers an upregulation of TSP4 in spinal cord and cortical astrocytes, thereby facilitating NP through the generation of new synapses and reorganization of somatosensory cortical circuits [220, 221]. In addition, the activation of sphingosine‐1‐phosphate receptor 1 (S1PR1) found on spinal astrocytes triggers the activation of nod‐like receptor family, pyrin domain containing 3 (NLRP3) inflammasomes. This leads to an endogenous increase in the synthesis and secretion of inflammatory cytokines, which subsequently disrupts glutamatergic homeostasis and enhances neuronal excitability. Furthermore, when the IL‐1β receptor is activated, it establishes a reciprocal mechanism that further amplifies both inflammatory cytokine production and sphingosine kinase (SphK) activity, thereby intensifying S1P levels [222].

Oligodendrocytes are responsible for creating the myelin sheath, which offers both structural support and electrical insulation to axons in the CNS system. The release of IL‐33 from spinal oligodendrocytes has been observed to potentially activate cellular signaling in spinal microglia, astrocytes, or endothelial cells, leading to the production of TNF‐α and IL‐1β. These cytokines then act on spinal neurons, facilitating synaptic transmission to exacerbate NP [223]. On the other hand, when oligodendrocytes are eliminated by toxins, symptoms of NP arise, suggesting a potential protective role played by these cells [224]. These findings indicate that oligodendrocytes actively participate in pain processes but their roles may vary depending on the specific context [28].

### 4.3 Epigenetic Modification

DNA methylation, histone modification, and noncoding RNAs (ncRNAs) play crucial roles in the modulation of NP [2]. DNA methylation and histone modification represent the prevalent covalent chemical alterations of chromatin to modulate gene expression at the transcriptional level. They have been shown to induce the reduction of opioid receptors and various K^+^ channels, as well as the upregulation of specific glutamate receptors, growth factors, and lymphokines [225]. ncRNAs also participate in the initiation and maintenance of pain perception. Generally, ncRNAs interact with mRNA, proteins, or DNA to modulate molecules and signals associated with ion channels, neuroinflammation, and neurotrophic factors among other biological processes. Consequently, they are involved in the progression of NP [226].

#### 4.3.1 DNA Methylation

Catalyzed by DNA methyltransferases (DNMTs), the transfer of methyl groups from S‐adenosylmethionine takes place at specific nucleotide bases. The majority of DNA methylation sites display aggregated distributions, commonly referred to as CpG (Cytosine–phosphate–Guanine) islands. Studies have demonstrated significant associations between DNA methylation and pain perception in NP conditions [2, 225]. Proteins regulated by DNA methylation in neuropathic conditions primarily include opioid receptors, K^+^ channels (Kv1.2, K2p1.1, Kv5.1), BDNF, glutamic acid decarboxylase 67 (GAD67), metabotropic glutamate receptors 5 and 4 (mGlu5, mGlu4), serotonin 5‐HT_4_ receptor, β2 adrenergic receptor, P2X3 receptor, and transcription factors SOX10 [225]. Significantly, DNA methylation exhibits disease and organ specificities [2]. For instance, significant variations in DNA methylation patterns can be observed between CINP, nerve injury‐, and diabetes neuropathy‐induced NP, despite their shared characteristic as typical forms of NP [2]. In the DRG, CpG sites tend to exhibit prevailing hypomethylation, whereas the CNS, including the spinal cord and prefrontal cortex, shows a higher degree of DNA methylation [2, 227].

The DNMT family primarily comprises three catalytically active enzymes, namely, DNMT1, DNMT3A, and DNMT3B, which play a role in the methylation of specific regions within genes. They typically display reduced methylation levels and contribute to the regulation of NP in both PNS and CNS [228]. DNMT1 and DNMT3A enhance the methylation process of the promoter and 5’‐untranslated region of the Kv1.2, resulting in a reduction in membrane densities of Kv1.2 channels and its current, thereby inducing NP [228]. On the other hand, ten‐eleven‐translocation proteins (TETs) play a crucial role in mediating DNA demethylation, thereby significantly contributing to the maintenance of DNA methylation stability and shaping the epigenome landscape. Significantly, the dual functionality of TET1 on pain has been unveiled [2].

#### 4.3.2 Histone Modification

Histone acetylation plays a pivotal role in regulating histone activity and primarily occurs at lysine residues on H3 and H4 [2]. In neuropathic models, hyperacetylation of H3 and H4 is enhanced by the inflammatory mediators IL‐6 and TNF‐α, leading to an increase in neuronal excitability [229, 230].

The equilibrium of histone acetylation is upheld through the activities of enzymes known as histone acetyltransferases (HATs) and histone deacetylases (HDACs) [2, 225]. Both HDAC1 and HDAC2 were found to be upregulated in the spinal cord response to injury, while the expression level of HDAC3 remained unchanged [225]. Recruitment of HDAC2 to the nucleus conversely exacerbates neuronal dysregulation and microglial‐related inflammation. This suggests that the subcellular distribution of epigenetic modulators plays a supplementary role in regulating pain [231]. Moreover, epigenetic inhibition of HDAC4 translocation into the cytoplasm leads to a reduction in high‐mobility group box 1 (HMGB1) expression, thereby serving as an analgesic strategy for NP [232]. Additionally, nuclear HDAC5 accumulation hinders H3 acetylation on the Gad1 and Gad2 promoters, thereby resulting in compromised activity of GABAergic neurons. This subsequently triggers abnormal activation of astrocytes via direct interaction with signal transducer and activator of transcription 3 (STAT3) [233, 234]. The pain‐relieving effects of silent information regulator (SIRT1 and SIRT3), which belong to class III of HDACs, were also been unveiled [2].

p300 serves as a prototypical mediator for pain modulation within the HATs family [2]. An increased binding of p300 to the COX‐2 gene's promoter region was observed, along with upregulation of the HAT p300 and overexpression of COX‐2 enzyme in the dorsal horn in neuropathic animals [225]. These findings indicate that nerve injury induces upregulation of COX‐2 through histone acetylation mediated by p300.

The histone methyltransferase EZH2, which catalyzes methylation of histone H3 at the K27 site, has been identified as a biomarker for assessment of the effectiveness of analgesic therapy in treating NP [2, 235]. Downregulation of EZH2 expression or the topical administration of EZH2 inhibitors has been observed to provide relief for neuropathic and cancer pain [236, 237]. The upregulation of EZH2 and H3K27 in microglia is induced by the enhanced expression of CGRP via a PKA and PKC pain pathway, following nerve injury [237]. Subsequently, microglia are activated, leading to a rapid release of proinflammatory factors such as IL‐1β and TNF‐α [236].

#### 4.3.3 Noncoding RNAs

ncRNAs involve a variety of RNA molecules that are primarily unable to encode proteins [2]. They possess the capacity to regulate neuronal excitability within the nociceptive pathway and influence neuroimmune interactions. In models of NP induced by nerve injury, dysregulation of ncRNA expression occurs in damaged nerves, DRG, spinal cord, and blood [238, 239]. NcRNAs, including microRNA (miRNA), long ncRNA (lncRNA), circular RNA (circRNA), and Piwi interacting RNA (piRNA), contribute to the development of NP [2, 21, 226, 240].

The main function of miRNAs is to bind to the 3′‐UTR regions of mRNA, which leads to the suppression of mRNA translation and promotion of mRNA degradation [2]. A significant quantity of target genes have been identified, involving inflammatory mediators, ion channels, transcription factors, transporters, and signaling molecules [226]. Importantly, the miRNA regulatory network on pain is highly intricate, with the additional complexity that one target gene can be regulated by multiple miRNAs [2].

LncRNAs and circRNAs can act as miRNA sponges by engaging in complementary base pairing, thereby suppressing the activity of miRNAs [2]. Mechanistically, lncRNAs and circRNAs play crucial roles in the regulation of gene expression related to pain through their interactions with RNA molecules, proteins, and DNA [241]. Additionally, the activity and subcellular localization of transcription factors can be influenced by its interaction between lncRNAs and circRNAs. Consequently, this leads to modifications in gene expression associated with NP [226]. Recent research has revealed the regulatory impact of piRNAs on NP [226]. Overexpression of spinal piR‐DQ541777 in mice elicited nociceptive responses and augmented neuronal sensitization through enhancing CpG island methylation [242].

### 4.4 Endoplasmic Reticular Stress

Endoplasmic reticulum (ER) serves as a primary site for the synthesis of secretory and membrane proteins. Misfolded/unfolded proteins accumulate within the lumen of the ER, leading to a state referred to as “ER stress” [243]. The latest research indicates that ER stress plays a pivotal role in initiating multiple molecular cascades associated with the development of chronic NP. The induction of ER stress can lead to various models of NP, including those induced by nerve ligation, metabolic dysfunction, vasculitis, multiple sclerosis, and stroke, as well as cancer and CINP [243]. Hyperexcitability in nociceptive neurons and increased pain perception can be triggered by ER stress, which operates through both inflammatory and noninflammatory pathways. ER stress can lead to dysregulation of multiple molecular cascades, such as mitochondrial functions, inflammatory responses, ROS generation, ion channel functions, calcium handling, and apoptosis [243]. Ultimately, the synergistic interplay among these disrupted pathways can lead to hyperalgesia. The application of various inhibitors targeting ER stress and unfolded protein response has demonstrated the alleviation of pain through simultaneous restoration of multiple molecular cascades [244, 245].

## 5 Molecular Mechanisms Of Nociplastic Pain Modulation

### 5.1 Central Sensitization

**FIGURE 4:**
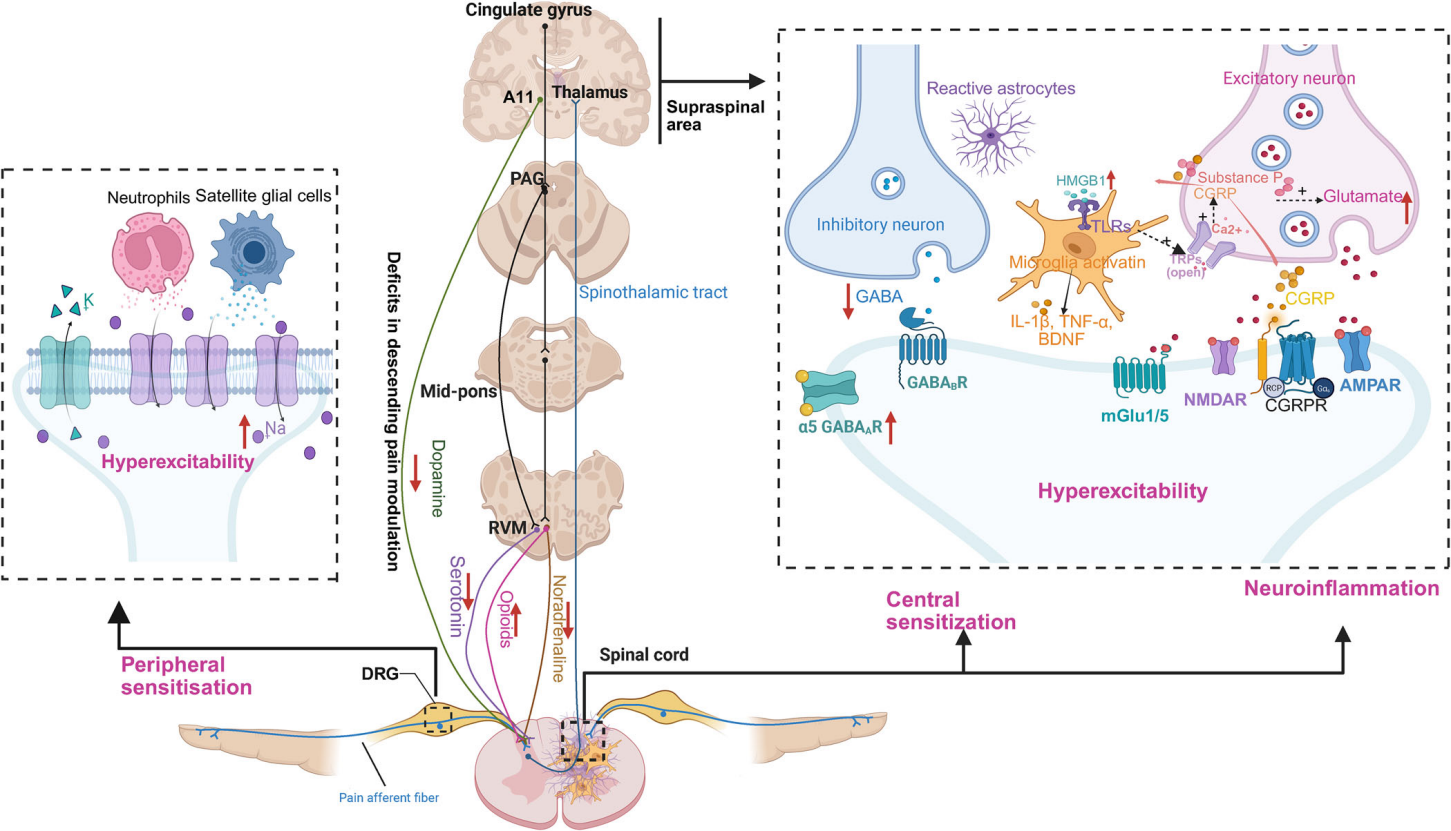
Neuroimmune mechanisms relevant to nociplastic pain syndromes in both the peripheral and CNS. Nociplastic pain syndromes involve a variety of complex pathophysiological mechanisms, primary including neuroimmune interactions, central sensitization, monoaminergic unbalance, and peripheral sensitization. The increased neutrophils and SGCs in sensory ganglia is crucial for the promotion of heightened pain sensitivity and sensitization of dorsal horn cells to harmful stimulation in nociplastic pain. The upregulation of pronociceptive molecules, such as TNF‐α, IL‐1β, and HMGB1, released from glial cells enhance neuroinflammation and pain signaling in CNS. For example, microglia TLR4 activation by HMGB1 contributes to microglia stimulation and neuroinflammation leading to the development of chronic nociplastic pain. Activation of TLR4 and TLR7 has been shown to enhance the activity of TRPV1 and TRPA1 channels, respectively, thereby facilitating neuronal excitability through enhancing Ca^2+^ influx, substance P as well as CGRP release. Enhanced excitatory signaling through glutamate and reduced GABA expression in the CNS contributes to central sensitization. Moreover, microglia release proinflammatory cytokines and pronociceptive neurotrophic factors into the CNS, thereby promoting the development of neuroinflammation and central sensitization. The descending pain modulatory system, involving endogenous opioids, noradrenaline, serotonin, dopamine, and endocannabinoid, plays an important role in the development of nociplastic pain. AMPA indicates a‐amino‐3‐hydroxy‐5‐methyl‐4‐isoxazolepropionic acid receptor; BDNF, brain‐derived neurotrophic factor; CGRP, calcitonin gene‐related peptide; CNS, central nervous system; GABA, gamma‐aminobutyric acid; α5‐GABA, α5 subunit containing GABA type A; HMGB1, high‐mobility group box 1 protein; IL‐1β, interleukin‐1 beta; NGF, nerve growth factor; NMDA, N‐methyl‐D‐aspartate receptor; PAG, periaqueductal grey; RVM, rostral ventromedial medulla; TNF‐α, tumor necrosis factor alpha; TLR, Toll‐like receptor; TRPV1, transient receptor potential vanilloid 1. The red arrow signifies an increase or decrease, while other‐colored arrows indicate secretion. The plus sign (+) signifies promotion.

The mechanisms underlying nociplastic pain remain incompletely understood; however, it is believed that increased sensitivity to pain in CNS and sensory information, coupled with changes in pain regulation, are considered significant factors [25]. Central sensitization pertains to the enhancement of nociceptive transmission in the dorsal horn of the spinal cord, leading to subthreshold sensory stimuli acquiring the capability to generate APs along pain pathways [24, 246]. Undoubtedly, central sensitization provides a mechanistic rationale for the manifestation of pain symptoms like allodynia and hyperalgesia in individuals experiencing nociplastic pain [24, 246]. The pathophysiology of central sensitization is characterized by enhanced excitatory signaling through glutamate and reduced GABA expression (as shown in Figure 4). Imbalances in excitatory and inhibitory neurotransmitters within spinal and brain regions have been demonstrated to be closely associated with the development of nociplastic pain in both animal models and patients experiencing this condition [24, 246]. A recent study has also demonstrated that an increased excitatory tone in the anterior insula, compared with inhibitory tone, is associated with hyperalgesia and clinical pain in patients with fibromyalgia [247]. Moreover, microglia release proinflammatory cytokines and pronociceptive neurotrophic factors into the CNS, thereby promoting the development of neuroinflammation and central sensitization (delayed rectifier) [246, 248].

The excitatory neurotransmitter plays a crucial role in the mechanisms underlying nociplastic pain [246]. Numerous investigations have demonstrated elevated levels of glutamate and glutamine, especially in the insula and posterior cingulate regions, which are commonly linked to increased severity of clinical pain in patients with fibromyalgia [249, 250]. A recent meta‐analysis found indications of increased levels of glutamate and glutamine in the insula and posterior cingulate regions among different types of chronic pain conditions, including migraine, fibromyalgia, temporomandibular disorder, irritable bowel syndrome (IBS), cLBP, and pelvic pain, when compared with control groups [24, 251]. Additionally, the effectiveness of ketamine in alleviating pain is apparent through both preclinical and clinical research [252, 253]. This collective evidence presented further supports the crucial role of glutamate in the establishment and maintenance of nociplastic pain [24, 246].

Disturbance of equilibrium between excitatory and inhibitory inputs in central sensitization highlights the pivotal role of GABA in the dysfunction of pain pathways. In this context, GABA levels exhibit a reduction in the brains of mice and rats experiencing pain resembling fibromyalgia [254, 255]. Clinical investigations support this narrative, as evidenced by the observed decrease in GABA levels in the anterior insular cortex of 16 patients with fibromyalgia [256]. However, the expression of α5‐GABA_A_ receptor, which is an extra synaptic receptor for GABA, shows an increase in a pain model resembling fibromyalgia [257]. Additionally, alleviation of reserpine‐induced fibromyalgia in rats can be achieved through the blockade of spinal α5‐GABA_A_ receptors [257]. These findings reinforce the key role of impaired GABAergic tone in nociplastic pain conditions.

### 5.2 Deficits in Descending Pain Modulation

The descending pain modulatory system (DPMS) governs neural activity within the spinal dorsal horn and exerts an impact on the transmission of nociceptive signals from peripheral regions to the CNS [24]. The DPMS comprises the ACC (Anterior Cingulate Cortex), hypothalamus, amygdala, periaqueductal grey (PAG), and RVM. Research has demonstrated that individuals with nociplastic pain exhibit impairments in DPMS‐mediated pain modulation, as indicated by decreased activity and functional connectivity within these brain regions [24]. The key neurotransmitters implicated in the DPMS involve endogenous opioids, noradrenaline, serotonin, and dopamine (as shown in Figure 4) [24, 246]. Patients with nociplastic pain exhibit reduced levels of noradrenaline, serotonin, and dopamine in the CSF [258], while the levels of endogenous opioids in the CSF are increased [259]. Additionally, individuals with fibromyalgia exhibit a reduction in the number or availability of μ‐opioid receptors in the brain [260]. These data suggest that those experiencing nociplastic pain may have elevated levels of endogenous opioids, consequently leading to hyperalgesia induced by endogenous opioids [260]. The biochemistry results align with clinical observations indicating the inefficacy or potential exacerbation of nociplastic pain by opioids [261]. Supporting this hypothesis further, preliminary findings indicate that the use of low doses of naltrexone, an antagonist for the μ‐opioid receptor, can effectively alleviate fibromyalgia [262].

The decrease in serotoninergic neurons corresponds to a reduction in serotonin levels observed in nociplastic pain paradigms within the spinal cord, highlighting the crucial involvement of serotonin in modulating nociplastic pain [263]. In reality, the administration of serotonin reuptake inhibitors results in an increase in serotonin levels and subsequently elicits analgesic effects in a fibromyalgia model [264]. Furthermore, administration of 5‐HT(2C) receptor agonists systemically demonstrates effective alleviation of muscular hyperalgesia in a myalgia model induced by reserpine [265]. These results accentuate the therapeutic potential of serotonin reuptake inhibitors and serotonin agonists in nociplastic pain [246]. It is worth noting that serotonin has both pain‐relieving and pain‐promoting effects in chronic pain, with its actions dependent on the specific receptors activated. This highlights the complex role of the serotonergic system in regulating pain [266].

The nociceptive information is also facilitated by noradrenaline through the descending pain pathways [267].

Various clinical studies highlighted the variations in noradrenaline levels detected in both serum and CSF among individuals with fibromyalgia, indicating its substantial role in the pathophysiology of nociplastic pain [258, 268, 269]. In line with this, animal models have presented additional findings indicating decreased levels of noradrenaline in the spinal cord and specific regions of the brain in experimental fibromyalgia models [263]. Notably, the effectiveness of dual reuptake inhibitors that act on both noradrenaline and serotonin systems (e.g., atomoxetine, duloxetine, and milnacipran) has been shown to relieve heightened sensitivity of muscles [270]. Additionally, a clinical study revealed that daily administration of duloxetine resulted in a reduction in pain severity among patients with IBS [271].

The disrupted reward circuitry in chronic pain states has been demonstrated by numerous studies. Individuals with fibromyalgia exhibit an abnormal dopamine response to pain in the striatum [272], which aligns with similar findings observed in individuals with cLBP [273]. Consistent findings from diverse investigations consistently highlight a decrease in dopamine concentrations in CSF and serum, accompanied by impaired functioning of presynaptic dopamine receptors among individuals with fibromyalgia [274]. Moreover, pharmacological manipulation of dopamine receptors leads to a reduction in hypersensitivity within a pain model resembling fibromyalgia [275, 276]. Additionally, it has been reported that the activation of dopamine D2 receptors effectively suppresses chronic migraine by inhibiting the positive feedback loop involving GluA2/ROS in both in vivo and in vitro experiments conducted on rats [277].

Retrograde signaling involves the generation of an endocannabinoid in response to postsynaptic activity, which then travels backward across the synapse and attaches to presynaptic CB1 receptors (CB1Rs), ultimately regulating the release of neurotransmitters through inhibition. As a result, this binding process usually leads to a reduction in pain pathway signaling within nociceptive neurons [278]. In addition, cannabinoids have the potential to facilitate the release of GABA and glutamate neurotransmitters in the PAG, leading to direct stimulation of descending inhibitory pain pathways and enhancing their antinociceptive properties [279, 280]. Reduced levels of anandamide in the CSF of migraine patients may also indicate an impairment of the endocannabinoid system within these patients [281]. However, there is evidence supporting elevated levels of circulating endocannabinoids among fibromyalgia patients [282].

In summary, nociplastic pain patients and animal models consistently exhibit elevated levels of opioids and decreased levels of serotonin, noradrenaline, and dopamine. Manipulating these neurotransmitters through various receptors shows promising therapeutic effects [24, 246].

### 5.3 Neuroinflammation

While nociplastic pain may not exhibit any discernible injury or inflammation, recent findings highlight the substantial involvement of immune system components in its underlying mechanisms [24, 246]. For instance, mice with reserpine‐induced fibromyalgia exhibited a significant rise in proinflammatory cytokines like TNF‐α and IL‐1β in their bloodstream, which correlated with the increased activity of NLRP3 inflammasome [254, 283]. In similar, in the acid saline‐induced fibromyalgia model, there was an elevation observed in levels of IL‐1β and IL‐6 [284]. Furthermore, administering IL‐10 into the muscles effectively reduced hyperalgesia after acid saline injections [285]. Despite this preclinical evidence, traditional treatments such as anti‐inflammatory or immunosuppressive therapies have proven ineffective in addressing classic nociplastic pain conditions like fibromyalgia, IBS, tension headache, and interstitial cystitis/bladder pain syndrome [246]. On the contrary, research on CSF samples from individuals with fibromyalgia indicates that inflammatory molecules and signaling pathways exhibit heightened activity in the CNS during nociplastic pain compared with healthy individuals [286, 287]. These findings indicate that sensitization through inflammatory signaling in CSF and CNS may serve as a potential pathophysiological mechanism for nociplastic pain conditions (as shown in Figure 4) [24, 288].

Microglial polarization and microgliosis are the primary hallmarks of neuroinflammation and serve as drivers of nociplastic pain [248]. Evidence from positron emission tomography studies suggests dysregulation of microglia and astrocytes in nociplastic pain conditions [289]. Indeed, activation of microglia through various neuromolecules, including ATP and Substance P, plays a vital part in the development of nociplastic pain [248, 290, 291]. The activation of microglial TLR has also been demonstrated to contribute to the stimulation of microglia and neuroinflammation, ultimately leading to nociplastic pain [292]. Increased glial activity in nociplastic pain is a potential mechanism by which TLR4 antagonists (like naltrexone) relieve pain in fibromyalgia, although regulation of endogenous opioidergic tone is also a plausible hypothesis [262].

TLR4 is typically found on sentinel immune cells, as well as astrocytes and microglia in the brain and spinal cord [246, 293]. TLR4 has been suggested to play a significant role in the development of nociplastic pain syndromes, such as fibromyalgia, stress‐induced, and sleep deprivation‐induced pain [246]. Reactive activation of microglia TLR plays a role in stimulating microglia and promoting neuroinflammation, ultimately resulting in the emergence of persistent nociplastic pain [248]. Indeed, the activation of TLR4 and TRL7 has been shown to enhance the activity of TRPV1 and TRPA1 channels, respectively, thereby facilitating neuronal excitability in individuals with fibromyalgia [246, 294]. The stimulation of TLR4 in whole blood is associated with elevated release of cytokines and chemokines in various pain syndromes, such as chronic fatigue syndrome, endometriosis, fibromyalgia, IBS, migraine, and low back pain [295, 296]. In addition, the activation of TRPA1 channels leads to the secretion of substance P and CGRP from peripheral terminals, thereby facilitating neurogenic inflammatory responses [297], which is presently acknowledged as a prominent mechanism underlying migraine [298].

### 5.4 Peripheral Sensitization

Peripheral mechanisms likely play a role in nociplastic pain, but their specific contribution to its development is still debated [24]. Injection of IgG from individuals with fibromyalgia into mice induced hyperalgesia and enhanced nociceptor responsiveness [299], although an independent research group failed to replicate this effect [300]. The results of subsequent investigations have demonstrated that IgG specifically attaches to anti‐SGCs in patients with fibromyalgia, and the extent of this attachment is correlated with the severity of the disease [301]. Meanwhile, a recent investigation has revealed that the presence of neutrophils in sensory ganglia is crucial for the promotion of heightened pain sensitivity and sensitization of dorsal horn cells to harmful stimulation in fibromyalgia. These effects were found to be reversible upon depletion of neutrophils, providing evidence for a mechanism in which CNS sensitization depends on neutrophil activity [300]. The consensus among these studies is that the DRG plays a potentially significant role in contributing to pain hypersensitivity observed in nociplastic pain models. Additionally, the involvement of sodium channel proliferation has been proposed to have a substantial impact on the development of nociplastic pain [7]. Furthermore, it is commonly observed that the sympathetic nervous system exhibits increased activity in conditions characterized by diffuse pain such as fibromyalgia and IBS [7]. Consistent with this, the interaction between inflammation and the autonomic nervous system can exacerbate endometriosis and is closely correlated with endometriosis‐associated pain [302].

## 6 Mixed Pain

One has to consider that pain is rarely exclusively nociceptive or neuropathic. This highlights the need for a continuum‐based classification system for pain [7]. Mixed pain involves a complex interplay between nociceptive, neuropathic, and nociplastic pain types, occurring simultaneously or concurrently within the same anatomical region. The relative dominance of each mechanism may vary over time, leading to either acute or chronic mixed pain [16]. However, some mixed pain may also indicate the existence of an entirely distinct pathophysiological mechanism [303]. It is increasingly recognized that many pain conditions, particularly those related to cancer and spine pain, exhibit a mixed pain phenotype. A large study estimated that the prevalence of mixed pain among patients with chronic pain is over 50% [16, 304]. Many professionals believe that the various forms of pain exist on a temporal continuum, with the main distinction between neuropathic and non‐NP being the lack of transduction in cases of NP [7]. This framework may provide an explanation for the occasional improvement of NP by NSAIDs, despite their primary efficacy in nociceptive pain [7, 305].

Currently, the identification of mixed pain relies on clinical assessment through a comprehensive evaluation of patient's medical background and detailed physical examination, rather than relying on explicit screening or diagnostic criteria. This absence of standardized screening or diagnostic criteria for mixed pain poses challenges for primary care physicians who frequently come across patients exhibiting potential mixed pain conditions in their everyday practice [303]. Furthermore, its pathophysiology remains elusive, resulting in the absence of established treatment guidelines at present. There is a growing interest within the medical community regarding the subject of mixed pain. Each patient exhibits unique responses to various situations, interventions, surgeries, and medications. This holds particularly true in cases involving mixed pain scenarios, where distinct pain pathways are implicated. The adjustment of treatments and diagnosis for each individual is therefore crucial. A personalized combination of the aforementioned treatments, tailored to meet the specific needs of each patient, should be implemented. [306]

## 7 Potential Therapeutic Strategies

The guidelines for managing chronic pain may vary depending on whether they pertain to symptom treatment (such as neuropathic or back pain) or a specific condition (like knee OA). Additionally, the perspective of the authors and their respective specialties (surgical or nonsurgical) can influence the content of guidelines [7, 307]. The optimal approach to pain treatment is mechanism‐based; however, in clinical practice, it can be challenging or even impossible to identify the underlying mechanisms of pain. Therefore, the management usually depends on approaches that target symptoms or diseases (as showed in Tables 2 and 3). For many patients, therapy goals should be individualized to focus on enhancing quality of life, which may be more attainable than achieving significant pain reduction [7]. Pain is a complex outcome resulting from various biological, psychological, and social factors; therefore, interdisciplinary treatment has been recommended in guidelines to optimize outcomes. Ideally, this approach should incorporate personalized strategies within a shared decision model [308, 309].

**TABLE 2:** Recommended medications for different types of pain.

Agents	Mechanisms	Disease
NSAIDs (celecoxib)	Inhibition of both COX‐1 and COX‐2, leading to a reduction in prostaglandin synthesis [310]; activation of CNS [311], anti‐inflammatory action is attributed to the inhibition of COX‐2 [312]	CPPP, OA, rheumatoid arthritis, traumatic pain, gout, other rheumatic disorders [313, 314, 315]
Paracetamol	Formation of the bioactive N‐arachidonoylphenolamine metabolite in the CNS to activate TRPV1 and Cav3.2 channel, thereby reinforcing the activity of descending serotonergic pathways [316]	CPPP, OA, headache, migraine attacks [316, 317, 318]
Tricyclic antidepressants (TCAs)	Hindering the reuptake process of serotonin and noradrenaline [2, 319]; modulation of opioid, glutamatergic and endocannabinoid systems, as well as blockade of sodium and calcium channels [2, 320]; anti‐inflammatory properties [321]	Postherpetic neuralgia, DN, central NP [20, 322]
Other antidepressants	SNRIs enhance serotonin and norepinephrine levels by inhibiting their reuptake; SSRIs inhibit serotonin reuptake [2, 323]	SNRIs are recommended as first‐line treatment for NP [20]
Ketamine	Antagonism of NMDAR [324, 325]; activating the DPMS and exhibiting anti‐inflammatory properties [2, 324]; its metabolite activate mTOR signaling and suppress CGRP release by inhibiting TRPV1 and TRPA1 channels in the CNS [326]	CPPP, NP, migraine, CRPS, fibromyalgia, phantom limb pain [324, 327]
Gabapentinoids	Binding to the α2δ subunit, thereby reducing ion influx; engagement of DPMS; anti‐inflammatory properties [2, 328]	DN, postherpetic neuralgia, spinal cord injury, [18, 329, 330] post‐total joint arthroplasty [331]
Lidocaine	Targeting Nav 1.7 and 1.8 on sensory afferents to impact both the generation and conduction of nerve impulses; activating TRPV1 and TRPA1 on keratinocytes and immune cells [332, 333]	CPPP, OA, DN, postsurgical pain, chronic carpal tunnel syndrome, lower back pain [332, 333]
Cannabinoids	Interaction with Nav1.7 and Nav1.8 to suppress neuronal activity; anti‐inflammatory properties; activating GABAergic neurons [334, 335]	NP, fibromyalgia, geriatric pain, cancer pain (a valuable adjunct or alternative) [279]
Opioids	Agonists on μ, κ, and δ receptors; activating inwardly rectifying potassium channels and inhibiting TRP channels, VGSCs, and ASICs; anti‐inflammatory properties [2, 336]	No longer recommended as a first‐line treatment for any type of chronic pain [337]
Tramadol	Activating opioid receptors in the CNS and inhibiting reuptake of norepinephrine and serotonin in the presynaptic site of the DPMS [2]	CPPP, chronic musculoskeletal pain, a secondary treatment option for NP [329, 338]
Capsaicin	Dephosphorylation and desensitization of TRPV1 by increasing permeability of TRPV1 to calcium; interaction with the high‐voltage‐activated calcium channel [339]	Polyneuropathies, DN, postherpetic neuralgia, HIV‐induced neuropathy [340]

Abbreviations: ASICs, acid‐sensing ion channels; CGRP, calcitonin gene‐related peptide; COX, cyclooxygenase; CPPP, chronic postoperative pain; CRPS, complex regional pain syndrome; DN, diabetic neuropathy; DPMS, descending pain modulatory system; mTOR, mammalian target of rapamycin receptor; NMDAR, N‐methyl‐D‐aspartic acid receptors; NP, neuropathic pain; OA, osteoarthritis; SNRIs, serotonin–norepinephrine reuptake inhibitors; SSRIs, selective serotonin reuptake inhibitors; TRPA1, transient receptor potential A1; TRPV1, transient receptor potential vanilloid 1; VGSCs, voltage‐gated sodium channel.

**TABLE 3:** Recommended nonpharmacological therapies for different types of pain.

Therapy	Mechanisms	Disease
Psychological strategies	Restructuring maladaptive beliefs, attitudes, and behaviors that contribute to disease burden [2, 336]; promoting global alterations in brain region activities [2]	cLBP, OA, IBS, temporomandibular disorders [2]
Virtual reality (VR)	Providing a distraction to effectively reduce pain [7]	cLBP, chronic neck pain, spinal cord injury [341, 342]
Exercise	Positive impacts on the peripheral nervous system through inhibiting neuronal degeneration, enhancing neurotrophic factors, and exerting antioxidant and anti‐inflammatory properties [343]; increasing the concentrations of endogenous opioids in the DPMS [2]	cLBP, OA, IBS, chronic musculoskeletal pain, DN, CIN, temporomandibular disorders [2, 7, 343]
Acupuncture	Anti‐inflammatory properties; reinstating the DPMS [2, 344]	Cancer, cLBP, OA, CPPP [2, 344]
Electrostimulations	Inhibition of nociceptive primary afferent fibers, downregulation of nociceptive neurotransmitters, and modulation of autonomic and inflammatory pain mediators [7, 345]	Various types of peripheral neuropathy, spinal cord injury, poststroke pain, CRPS, fibromyalgia, migraine, cluster headache, visceral pain (abdominal pain due to menstruation) [7, 345, 346]

Abbreviations: CIN, chemotherapy‐induced neuropathy; cLBP, chronic low back pain; CPPP, chronic postoperative pain; CRPS, complex regional pain syndrome; DN, diabetic neuropathy; DPMS, descending pain modulatory system; IBS, irritable bowel syndrome; OA, osteoarthritis.

### 7.1 Treatments for Nociceptive Pain

The first‐line treatments for nociceptive pain include topical and oral NSAIDs. Compared with NSAIDs, acetaminophen (paracetamol) does not possess anti‐inflammatory properties. The efficacy of muscle relaxants has been demonstrated in the treatment of acute spinal pain, but there is limited evidence supporting their use for chronic neck or back pain [347]. Scant evidence exists regarding the effectiveness of benzodiazepines among different muscle relaxants [347], as they can lead to physical dependence and elevate the likelihood of complications associated with opioids [348]. The analgesic antidepressants have demonstrated efficacy in the treatment of low back pain, despite being specifically indicated for NP [347].

Among the numerous available NSAIDs, ibuprofen, diclofenac, and ketoprofen continue to be the most commonly utilized [349, 350]. Primary indications for the use of NSAIDs include CPPP, acute arthritis, traumatic pain, RA, and various other disorders affecting the joints and connective tissues. It should be noted that not all NSAIDs possess identical characteristics. Specifically, when selecting the appropriate NSAIDs for an individual patient, it is crucial to consider medication‐related factors (such as pharmacokinetic properties) as well as patient‐related characteristics (including comorbidities, and potential adverse effects) [349].

Corticosteroids are frequently used in the treatment of inflammatory pain [351]. According to the EULAR guidelines, corticosteroids are recommended as a viable and efficient option for pain management associated with crystal‐induced arthritic conditions in cases where NSAIDs are poorly tolerated [352, 353]. Furthermore, platelet‐rich plasma therapy has been demonstrated to effectively alleviate various types of OA pain by promoting chondrocyte proliferation and facilitating the development of cartilage matrix, while inhibiting the production of inflammatory factors [354, 355].

Biologic drugs may be beneficial in the management of nociceptive pain. Anakirna, an antagonist of IL‐1 receptors, demonstrated a significant reduction in gout pain [356]. The IL‐1 inhibitors rilonacept and canakinumab have also demonstrated efficacy in reducing the inflammatory pain associated with gout [357]. The involvement of NGF in pain signaling associated with OA has been observed, suggesting its potential role [349]. Furthermore, the United States Food and Drug Administration (US FDA) has granted approval for tanezumab, a humanized monoclonal IgG2 antibody that effectively inhibits the binding between NGF and TrkA receptors, specifically for treating OA and lower back discomfort [358]. The recombinant fully human anti‐NGF antibody, fasinumab, is currently undergoing clinical trials and holds promising potential as a therapeutic intervention for OA [352]. NTP is commercially available for the management of chronic painful conditions related to inflammation in Asia. Its mechanism involves the suppression of descending pain pathways and inhibition of inflammatory signaling and cell death pathways [349, 359].

The natural antimitotic alkaloid, colchicine, is widely utilized in the treatment of gout [349]. Colchicine can effectively alleviate pain and inflammation associated with crystal‐induced arthritis, which is characterized by intense inflammatory processes triggered by the deposition of crystals in synovial tissues [360, 361, 362].

Overall, the treatment of nociceptive pain needs to take into account the type of pain, the specific situation of the patient, and the degree of inflammatory response to formulate an individualized treatment plan. Furthermore, treatment strategies should be continuously updated and optimized as our understanding of the mechanisms behind nociceptive pain deepens.

### 7.2 Treatments for NP

The management of NP primarily focuses on symptomatic treatment due to the limited efficacy in addressing the underlying cause; moreover, addressing etiological conditions such as diabetes mellitus often fails to provide adequate relief from NP. Patients with NP typically exhibit poor responsiveness to analgesics like acetaminophen, NSAIDs, or weak opioids. The conventional approach to managing NP in patients involves commencing treatment with conservative pharmacological and complementary therapies prior to considering the implementation of interventional techniques like nerve blocks and neuromodulation [18].

The first‐line medications for NP are analgesic antidepressants and antiepileptic drugs. Pregabalin and gabapentin have emerged as the preferred treatment options and have received US FDA approval for managing NP associated with DN, spinal cord injury, and postherpetic neuralgia [18, 329, 330]. Gabapentinoids have demonstrated efficacy primarily in two distinct types of NP: postherpetic neuralgia (with a minimum 50% reduction in pain reported by 41% of patients for pregabalin and 32% of patients for gabapentin) and DN pain (with a minimum 50% decrease in pain reported by 41% of patients for pregabalin and 38% of patients for gabapentin) [363, 364]. Other studies have demonstrated a moderate impact of gabapentinoids on alleviating NP caused by spinal cord injury, while their impact on central NP and HIV‐induced NP remains contradictory [364, 365]. However, limited research has been conducted to evaluate their efficacy in other forms of NP [366]. Pregabalin also demonstrates superior effectiveness in managing post‐total joint arthroplasty compared with gabapentin [331]. In contrast, the PROSPECT guidelines suggest caution or limited endorsement for gabapentinoids in managing various other forms of postoperative pain due to their unfavorable risk‐benefit ratio [367].

Among the various classes of antidepressant medications, TCAs (such as nortriptyline hydrochloride and amitriptyline hydrochloride), as well as SNRIs (such as duloxetine hydrochloride and venlafaxine hydrochloride) are indicated for the treatment of NP [7, 330, 368]. Among TCAs, amitriptyline is the most commonly prescribed medication for the treatment of postherpetic neuralgia, DN pain, and central NP [369]. Among SNRIs, duloxetine and venlafaxine are primarily utilized for DN and are endorsed by the NeuPSIG and European Federation of Neurological Societies as first‐line therapies for NP [366]. The overall effect of SSRIs is to suppress pain; however, clinical trials have demonstrated that the efficacy of SSRIs is comparatively lower than that of TCAs or SNRIs, potentially due to receptor interactions counteracting their effects [370]. According to the research, SSRIs only alleviate pain in approximately 14% of patients suffering from chronic NP [322]. Their extensive pharmacological effects increase the likelihood of potential adverse reactions. As a result, SSRIs are generally not used as the primary medications in clinical pain management [2].

Oxcarbazepine and carbamazepine are considered effective in treating NP, particularly as a first‐line treatment for trigeminal neuralgia. Studies have shown that carbamazepine can reduce pain by at least 30% in 88.3% of patients, while oxcarbazepine achieves similar results in 90.9% of patients [371, 372]. They are inhibitors of Nav channels and have demonstrated the ability to suppress spontaneous ectopic activity triggered by nerve injuries [366].

Botulinum toxin type A (BoNT/A), a protein known for its neurotoxic properties, is employed as a tertiary treatment option for NP in patients who exhibit resistance to other therapeutic options. Both single and repeated subcutaneous administrations of BoNT/A are utilized to manage conditions such as trigeminal neuralgia, postherpetic neuralgia, DN, and refractory NP including poststroke pain and spinal cord injury [373].

There is a rising interest in immunomodulation as an innovative therapeutic approach for individuals suffering from NP [195]. In certain circumstances, intravenous immunoglobulin treatment, which dampens immune responses, may provide relief for individuals suffering from chronic inflammatory demyelinating polyneuropathy‐associated pain [374]; however, it does not provide relief for those experiencing pain related to postpolio syndrome [375]. Moreover, supplementation of omega‐3 fatty acids resulted in a reduction in pain sensitivity and inhibited the functioning of inflammatory and oxidative stress pathways among patients with type 2 diabetes [376, 377]. Patients with different NP conditions, including cervical radiculopathy, thoracic outlet syndrome, carpal tunnel syndrome, fibromyalgia, and burn injuries, also experienced significant pain reduction for a duration of up to 19 months after starting daily omega‐3 supplementation. No adverse effects were observed during the treatment period [378]. Multiple clinical trials have also demonstrated the efficacy of IL‐6 receptor inhibitors like tocilizumab, and TNF inhibitors including adalimumab, etanercept, and infliximab in alleviating pain among individuals diagnosed with lumbosacral radiculopathy and sciatica [379, 380, 381]. However, a comprehensive examination and meta‐analysis revealed that the existing data were inadequate to endorse the utilization of anti‐TNF agents in individuals suffering from sciatica [382]. In all, these results underscore the positive impact of immunomodulatory therapies in certain NP syndromes but not universally across all types [195].

An increasing number of clinical studies have been focusing on novel pharmacological targets, such as ion channels and glutamatergic receptors. With respect to Nav1.8, new substances have been discovered that exhibit selective inhibition of this target to address NP. A clinical study further corroborated these findings by demonstrating the analgesic effects of a selective Nav1.8 inhibitor, VX 150 (Phase I), in patients with NP [366, 383]. A TRPA1 antagonist, GRC 17356, also demonstrated promising results in a Phase II clinical trial for the treatment of DN [384]. In addition, various P2X receptor antagonists have undergone or are presently undergoing clinical assessment for various forms of NP. For instance, clinical trials are currently underway to investigate the efficacy of P2X4 receptor antagonists like NP‐1815‐PX and NC‐2600 in addressing NP conditions [366].

Consequently, the effective management of NP requires a holistic approach that integrates diverse therapeutic interventions. Opioids, such as morphine and oxycodone, are typically reserved for severe cases; however, their use is associated with certain risks [385, 386]. Tramadol and tapentadol are analgesics that act on the CNS and are commonly used to alleviate moderate to severe NP [387]. Each of these therapeutic options presents distinct advantages and considerations, and the selection of second‐line treatments should be guided by individual patient factors [320].

### 7.3 Treatments for Nociplastic Pain

Recommending a multimodal and stepwise approach based on severity, similar to the one suggested by the US Veteran's Health Administration, is considered ideal for addressing nociplastic pain. This involves starting with education and self‐care interventions, gradually advancing to more sophisticated therapies if deemed essential [25, 388]. The management strategies for nociplastic pain aim to attenuate symptoms rather than eradicate them, with a primary focus on nonpharmacological treatments as the preferred initial approach that can be tailored individually (precision medicine). The treatment goals should not only focus on pain relief but also include improvement of function and other indicators related to quality of life [24, 25].

To date, there is a lack of established recommendations for managing nociplastic pain. The efficacy of traditional analgesic medications, such as NSAIDs and paracetamol, is typically limited in individuals with nociplastic pain. Opioids should be avoided in these cases [25]. The use of NSAIDs is warranted solely when there is clinical evidence of inflammation [279]. More potent medications include SNRIs like duloxetine and milnacipran, tricyclic compounds like cyclobenzaprine, noradrenaline reuptake inhibitors like esreboxetine, and gabapentinoids. The efficacy of duloxetine as a treatment for various chronic pain conditions, such as fibromyalgia and chronic headaches, has been demonstrated [389, 390]. The overall evidence regarding the efficacy of cannabinoids for musculoskeletal pain treatment remains inconclusive, whereas available data strongly support their use in managing fibromyalgia pain [279]. Studies indicate that ketamine treatment demonstrated a significant reduction in short‐term pain among patients suffering from chronic noncancer pain conditions, such as migraine, fibromyalgia, and complex regional pain syndrome. However, the long‐term effectiveness of ketamine in pain management remains uncertain [391].

The use of opioid analgesics is strongly discouraged [25, 392]. In addition to the recognized hazards linked with opioid treatment, individuals experiencing nociplastic pain may exhibit reduced sensitivity toward opioids due to elevated levels of naturally occurring opiates, exacerbation of hyperalgesia, and disruption in sleep patterns [393, 394]. In fact, the effectiveness of low‐dose naltrexone, an opioid antagonist that may increase the number of opioid receptors and improve the body's reaction to natural pain‐relieving substances, has been proven in the treatment of complex regional pain, chronic back pain, and fibromyalgia [262].

### 7.4 Opioids

Opioids are analgesic medications commonly employed across all age groups. These drugs play a crucial role in clinical symptomatic and palliative therapies. Opioids act as agonists on μ, κ, and δ receptors, exhibiting diminishing effects across these receptor subtypes. The clinical and social issues arising from opioids are primarily attributed to their adverse effects, such as tolerance, hyperalgesia, respiratory depression, and gastrointestinal response [2]. The current consensus is that opioids are no longer recommended as the initial treatment option for any form of persistent pain, and in certain populations (such as young individuals with noncancer pain), many guidelines do not recommend their use at all [337]. The VA/DoD guideline development group asserted that the potential for severe opioid‐related harms and significant adverse incidents, particularly in cases of prolonged usage, outweighed any potential benefits derived from temporary improvements in pain severity and functional status among patients with chronic pain [337, 395].

The weak opioid agonist tramadol is widely used for pain management, particularly in cases of postoperative and chronic musculoskeletal pain. Both CPS and EFNS guidelines also recommend tramadol as a secondary treatment option for NP [329, 338]. Furthermore, it can be employed with utmost safety and efficacy for pain relief without any adverse impact on the respiratory function of the neonate. Recently, a chewable tablet has been developed and employed for pediatric use, further substantiating its safety [396].

The updated 2022 VA/DoD guideline recommends prescribing buprenorphine as an alternative to full opioid agonists for individuals who are currently receiving daily opioids for the management of persistent pain [337]. The primary appeal of buprenorphine lies in its pharmacological properties, specifically its partial opioid agonism, and frequent coformulation with naloxone. This unique combination enhances safety by reducing the risk of misuse and overdose compared with full opioid agonists [397]. However, the recommendation for buprenorphine is based on evidence of limited quality [337].

### 7.5 Gut Microbiota

Accumulating evidence suggests the existence of a complex network of interconnections between gut microbiota and the CNS, known as the gut microbiota‐brain axis [398, 399]. The gut microbiota is capable of producing neurotransmitters (including GABA, serotonin, noradrenaline, and dopamine), metabolites (like short‐chain fatty acids [SCFAs] and bile acids), as well as PAMPs (such as lipopolysaccharide [LPS] and peptidoglycans). These products exert an impact on resident neuronal and immune cells in the gastrointestinal tract, thereby modulating signal transmission within vagal afferent pathways. They also traverse the intestinal barrier, gain access to the systemic circulation, and penetrate the CNS to interact with immune and glial cells. This interaction leads to the secretion of cytokines and chemokines to regulate nociceptive signaling in both peripheral and CNS [195, 398].

Currently, there are mainly preclinical animal studies suggesting that gut microbiota could potentially have a significant impact on nociceptive pain. Inflammatory hypernociception was observed to be attenuated in germ‐free mice. Reduction of hypernociception was associated with a decrease in tissue inflammation and could be reversed through the repositioning of the microbiota or subcutaneous injection of LPS. Importantly, observed reduction in pain hypersensitivity among germ‐free mice was linked to an increased expression of IL‐10 and could be reversed by using an anti‐IL‐10 neutralizing antibody [400]. Additionally, injection of monosodium urate monohydrate crystals resulted in joint inflammation, hyperalgesia, and increased levels of IL‐1β and CXCL1. These effects were significantly reduced in germ‐free mice and mice subjected to antibiotic treatment [401]. These findings suggest that targeting the gut microbiota or applying specific probiotics holds promise in mitigating pain hypersensitivity across various inflammatory conditions. In fact, probiotics containing lactic acid‐producing bacteria have been shown to alleviate pain in RA patients, concurrently leading to a reduction in proinflammatory cytokine levels within the serum [402, 403].

In nerve‐injured rodents with NP, dysbiosis of the microbiota was found to be correlated with altered levels of various metabolites in both serum and spinal cord. These metabolites primarily play a role in regulating lipid metabolism and the inflammatory response [195, 404]. The mediators derived from the microbiota have the ability to impact innate immune cells residing in the DRG and spinal cord, thereby exerting regulatory control over neuronal excitability and pain perception [398, 405]. The resolution of CINP may be attributed to the reduction of inflammatory factors via the LPS–TLR4 pathway in macrophages, which is facilitated by antibiotic treatment targeting gut microbiota eradication [406, 407]. Gut microbiota has also been implicated in regulating microglia in models of NP. Depletion of gut microbiota can alleviate thermal hyperalgesia by inhibiting the activation of spinal glial cells in animals with nerve injury or DN [406]. A separate investigation found that SCFAs derived from microbiota were involved in the regulation of microglial activation and the promotion of proinflammatory microglial polarization in mice with nerve injury. Antibiotic administration reversed pain sensitivity, reduced microgliosis, and suppressed the secretion of proinflammatory cytokines in the spinal cord and hippocampus following nerve injury. Interestingly, these beneficial effects were blocked when SCFAs were administered [408]. However, there is currently a limited repertoire of validated clinical approaches available for manipulating the gut microbiota in patients suffering from NP.

The involvement of gut microbiota in the pathogenesis of nociplastic pain, such as IBS, migraine, and fibromyalgia, has been established through preclinical studies. For instance, alteration of gut microbiota composition by means of antibiotic usage is correlated with an elevated susceptibility to IBS [409]. Additionally, it has been demonstrated that fecal transplantation from a human suffering from migraines induces nitroglycerin hyperalgesia in mice, thereby suggesting a potential association between the gut microbiota and susceptibility to migraines [410]. These studies have provided valuable insights into the mechanisms underlying the regulation of nociplastic pain by gut microbiota. Findings from clinical studies further support the notion. The antibiotic rifaximin effectively alleviates abdominal pain in patients suffering from IBS [411]. Specific probiotics demonstrated efficacy in alleviating abdominal pain among both adult and pediatric patients with IBS [412, 413]. Supplementation with probiotics demonstrated an improvement in the quality‐of‐life scores among migraine patients [414]. Additionally, foods low in monosaccharides, fermentable oligosaccharides, disaccharides, and polyols have been shown to alleviate visceral nociception in patients with IBS caused by gut dysbiosis and TLR4‐dependent mast cell activation triggered by LPS [415, 416]. A clinical case study of gut microbiota transplantation in patients with fibromyalgia also demonstrated significant symptom improvement and notable alterations of the enteric microbiota [417].

### 7.6 Nanomedicine

Nanomedicine involves the use of nanocarriers, specifically nanoparticles, to facilitate targeted drug delivery within the human body to previously inaccessible locations. At present, organic materials such as liposomes represent the primary application of nanoparticles in medical practice [352]. Nanomedicine has demonstrated enhanced efficacy of poorly soluble drugs with reduced dosages, attributed to the particles’ ability to enhance drug bioavailability [418]. These drug delivery systems employ specific parameters to optimize the circulation time of a drug and enhance its tissue/organ specificity [419]. Additionally, nanomedicine nanomedicine also showcases its potential to safely administer therapeutic doses of typically toxic substances with minimal local or systemic harm [352, 420, 421].

The utilization of nanomedicine for precise targeting of sodium channels represents an innovative and highly significant approach in the field of pain management [422]. Indeed, through the utilization of nanoscale carriers such as liposomes, adeno‐associated viral (AAV) vectors, and polymeric or lipid nanoparticles, NaV blockers can be precisely delivered to specific nerves or tissues with unparalleled precision [422]. Currently, nanomedicines targeting VGSCs have primarily been confined to local infiltration analgesia and peripheral nerve block for localized or regional anesthesia in surgical interventions as well as for the management of pain following surgery [422]. In addition, the versatility of nanomedicine also allows for the development of combination therapies that simultaneously target multiple pain pathways [423, 424]. For instance, it is possible to design nanoparticles that can transport both an anti‐inflammatory and an analgesic substance, effectively managing inflammation and pain in a complementary way. This strategy exhibits significant promise for improving pain alleviation in intricate conditions [422, 424].

### 7.7 Stem Cell Therapy

The field of stem cell therapy is a crucial component within the realm of regenerative medicine. Adult stem cells can be classified based on their source tissue, such as those obtained from the hematopoietic stem cells, placenta and umbilical cord, mesenchymal cells (MSCs) derived from bone marrow, and adipose tissue‐derived MSCs [421]. Their general mechanisms underlying the modulation of chronic pain include the following three aspects. The direct approach involves the rapid division of lesions and preparation of injured tissues. Stem cells also secrete a variety of trophic factors such as VEGF, transforming growth factor, and other anti‐inflammatory cytokines. Additionally, stem cells can globally modulate the immune system, including suppressing the differentiation of monocytes into dendritic cells, inhibiting T cell maturation, and regulating NK cell activity [2].

In preclinical studies, stem cells obtained from bone marrow, adipose tissue, and peripheral nerves have demonstrated significant attenuation of NP, such as sciatic nerve injury and DN [421, 425]. Transplantation of stem cells effectively reverses opioid tolerance [426]. VEGF serves as a potent regulator in the modulation of complex neuropathy. Enhanced pain relief is observed in patients with Parkinson's disease when treated with bioengineered stem cells that express VEGF [427]. The limited number of human trials notwithstanding, the available evidence effectively showcases the advantageous facets of stem cell applications. For example, treatment with MSCs has been shown to significantly alleviate pain in patients suffering from spinal cord injury and neuropathic facial pain [428, 429]. MSC therapy could also potentially serve as a viable alternative treatment option for individuals suffering from chronic back pain. After undergoing autologous expanded bone marrow MSC injection treatment, patients diagnosed with lumbar disc degeneration and suffering from chronic back pain experienced rapid improvement in both pain and disability, reaching 85% maximum improvement within a period of 3 months [430].

### 7.8 Gene Therapy

With the European Medicines Agency and the US FDA approving more than a dozen gene therapy programs out of over 800 in development, personalized medicine for various diseases has been significantly enhanced by the emergence of gene therapy [431, 432]. The methodologies of gene therapy can be classified into two categories according to the delivery systems: viral infection and nonviral vectors [2].

Ion channels are the main targets in gene therapy by viral delivery systems. Pain responses in mice were attenuated through the suppression of NaV1.7 in nociceptors mediated by clustered regularly interspaced short palindromic repeats (CRISPR)–dCas9 [433]. The study demonstrated that intrathecal infusion of CRISPR–dCas9 adeno‐associated virus 9 (AAV9) effectively attenuated inflammatory pain and NP [433]. A study was conducted to investigate the genetic variations of TRPV1, where a K710N missense variant of TRPV1 was introduced using CRISPR/dCas9 technology. This resulted in a reduction in calcium influx and a decrease in neuronal excitability, thereby suppressing nociceptive and NP [434]. In a mouse model of inflammatory hyperalgesia, the CRISPR–Cas9 editing of the PKC phosphorylation residue S801 in TRPV1 effectively alleviated masseter muscle inflammation‐induced pain, while preserving the physiological functions of TRPV1 [435]. In addition, delivery of the gene encoding CBD3 via AAV in DRG effectively alleviates NP through inhibiting the activity of Cav2.2 [436].

Naked plasmids offer a potent therapeutic strategy for the treatment of ischemic diseases [2]. Topical application of naked HGF plasmids effectively mitigates macrophage infiltration in the DRG, thereby reducing the secretion of proinflammatory factors, such as TNF‐α, IL‐1β, and IL‐6 [437, 438]. This pain relief strategy has successfully completed phase III clinical trials for DN [439], making it the most advanced gene therapy approach currently available for clinical application. Similarly, administration of naked plasmids containing anti‐inflammatory IL‐10 induces long‐lasting NP and OA‐pain suppression [440, 441]. Taken together, the concurrent development of dual‐track gene therapies has paved the way for future investigations in a novel direction.

## 8 Conclusion And Perspective

The current therapy of assessment and management for chronic pain still falls significantly short of adopting a mechanism‐oriented or precision medicine strategy. The newly introduced 2021 IASP clinical criteria and grading system for nociplastic pain facilitate prompt recognition and categorization of individuals according to their unique pain characteristics, representing a crucial advancement in the field of precision pain medicine [11, 442]. The present review elucidates the fundamental molecular mechanisms underlying nociceptive, neuropathic, and nociplastic pain, respectively. Immune responses and alterations in ion channels play a crucial role in the development of nociceptive and NP. Epigenetic modifications and endoplasmic reticular stress can further exacerbate NP, while the emergence of nociplastic pain is likely influenced by central sensitization, impairments in descending pain modulation, and neuroinflammation. Significantly, despite the emphasis on precision medicine, there is a lack of practical frameworks for integrating molecular profiling—such as biomarkers and genetic testing—into clinical workflows. Overlapping molecular pathways among various pain subtypes (e.g., shared neuroinflammation in neuropathic and nociplastic pain) affect therapeutic approaches. A thorough analysis of these cross‐subtype interactions is essential for refining the application of precision medicine.

In the part of therapeutic strategies, we reviewed some recent advancements in both pharmaceutical and nonpharmaceutical approaches to managing chronic pain. The first‐line treatments for nociceptive pain include topical and oral NSAIDs. In order to devise a personalized treatment plan, it is imperative to consider the type of pain, the specific circumstances of the patient, and the extent of inflammatory response. The conventional approach to managing NP in patients entails initiating treatment with conservative pharmacological and complementary therapies prior to employing interventional strategies. The management strategies for nociplastic pain primarily focus on individualized nonpharmacological treatments as the preferred initial approach. All in all, the guidelines for managing chronic pain may vary depending on whether they pertain to symptom management or a specific medical condition, ultimately leading to diverse approaches to treatment. Emerging treatment strategies, such as microbial intervention, nanomedicine, stem cell therapy, and gene therapy, have the potential to enhance the precision of chronic pain management. Meanwhile, tracking the ongoing clinical trials related to chronic pain treatment targeting ion channels will provide further direction for therapeutic management (Table 4).

**TABLE 4:** Clinical updates of potential ion channels modulators for chronic pain management.

Target channel	Drugs	Indications	Current status
Channel	GDC‐0276/GDC‐0310	Healthy volunteers	Phase I
	DSP‐2230/ANP‐230	Peripheral neuropathy	Phase I
	AZD‐3161	Ultraviolet exposed skin	Phase I
	NeoSTX	Local anesthetic	Phase I
	ST‐2427	CPPP	Phase I
	PF‐05089771	DN, dental pain	Phase II
	Funapide	PN, OA	Phase II
	Ralfinamide	cLBP	Phase III
	Vixotrigine/raxatrigine	Trigeminal neuralgia, cLBP	Phase III
Nav1.8 [422, 443]	PF‐06305591	Cold pain, inflammatory pain	Phase I
	PF‐04531083	Dental pain, ultraviolet exposed Small fiber neuropathy, OA	Phase I/II
	VX‐150		Phase II
Nav1.1/1.2/1.5	Halneuron	Cancer pain, CINP	Phase III
Nav1.8/ /1.9 [422]	Ambroxol	NP	Phase III
CaV2.2 [443]	Leconotide/CNSB004	Intractable pains	Phase IIa
	Z‐160	cLBP	Phase II
	CNV‐2197944	PN, DN	Phase II
CaV3.2 [443]	Z‐944	NP	Phase I
	ABT‐639	DN	Phase II
ASIC [109]	Amiloride	Migraine	Pilot study
	PPC‐5650	IBS, esophageal pain	Phase I
P2X3 [443]	Sivopixant	NP	Phase I
	Eliapixant	DN	Phase II
	Gefapixant	Endometriosis, OA	Phase II
	Minodronate	cLBP	Pilot study
P2X7 [129]	AZD9056	Chronic abdominal pain	Phase II
TRPV1 [443]	Resiniferatoxin	Cancer pain	Phase I
	NEO‐6860	OA	Phase II
	DWP‐05195	PN	Phase II
	Civamide/zucapsaicin	Episodic cluster headache	Phase III
TRPV3 [443]	GRC‐15300	NP	Phase II
TRPA1 [443]	HX‐100	DN	Phase I
TRPM8 [443]	Menthol	DN	Phase I/II
	PF‐05105679	Cold pain, NP	Phase I/II
	AMG‐333	Migraine	Phase I

Abbreviations: ASICs, acid‐sensing ion channels; Cavs, voltage‐gated calcium channels; CINP, chemotherapy‐induced neuropathic pain; cLBP, chronic low back pain; CPPP, chronic postoperative pain; DN, diabetic neuropathy; IBS, irritable bowel syndrome; NP, neuropathic pain; P2X, ionotropic purinergic receptors; PN, postherpetic neuralgia; TRPA1, transient receptor potential A1; TRPM8, transient receptor potential (TRP) transient receptor potential melastatin 8.; TRPV, transient receptor potential vanilloid.

To sum up, we have examined some innovative and contentious advancements in mechanical and therapeutic strategies that may offer new perspectives for the future management of chronic pain. The majority of recent preclinical and clinical investigations within the pain field have primarily centered around elucidating its underlying mechanisms and exploring therapeutic interventions. However, there are certain limitations that need to be addressed, such as insufficient research evidence, limited accuracy in experimental models, underutilization of omics data, and slow progress in the clinical translation of pain research [2]. The improvement and validation of reproducibility and predictability in animal models and outcome measures should be explored for the translation of preclinical findings to human pain conditions. A more comprehensive comprehension of the ligand‐binding sites, protein structure, and precise mechanisms of action of different analgesics has the potential to expedite pharmaceutical discovery. In this regard, the utilization of the AlphaFold Protein Structure Database and other artificial intelligence methodologies presents opportunities for drug innovation, as well as the selection and prioritization of drug target combinations. Moreover, it has the potential to improve the design of clinical trials and offer valuable perspectives on individualized treatment approaches [444, 445]. Herein, we propose several future perspectives for pain research, including the development of advanced experimental models, comprehensive application of omics, identification of biomarkers for chronic pain, elucidation of the mechanisms underlying the transition from acute to chronic pain, the development of nonopioid analgesics, and the exploration of brain mechanisms based on pain resilience.

## Author Contributions

Zhen Li, Xing Li, Jieqiong Liu, Rao Sun, Yingze Ye, Hongbing Xiang, Fang Luo, Shiyong Li, and Ailin Luo made significant contributions to the content discussion and actively participated in writing, reviewing, and editing the manuscript prior to its submission. The final version of the manuscript was approved by all authors.

## Conflicts of Interest

The authors declare no conflicts of interest.

## Ethics Statement

No ethical approval was required for this study.

## Data Availability

The authors have nothing to report.
